# PbrIDD2‐ *PbrAPX14* Module Functions in the Ethylene‐Mediated Ripening and Senescence Process of Pear Fruit

**DOI:** 10.1111/pbi.70664

**Published:** 2026-04-27

**Authors:** Libin Wang, Junpeng Niu, Xiaoyu Tan, Yuhua Xie, Li Jiang, Chen Huan, Xu Zhang, Weiqi Luo, Bing Xie, Xiaopu Ren, Guodong Wang, Yuanyuan Guo, Shaoling Zhang

**Affiliations:** ^1^ College of Horticulture Nanjing Agricultural University Nanjing Jiangsu China; ^2^ School of Food and Biological Engineering Hezhou University Hezhou China; ^3^ College of Light Industry and Food Engineering Nanjing Forestry University Nanjing Jiangsu China; ^4^ College of Life Sciences, Engineering Research Center of High Value Utilization of Western China Fruit Resources, Ministry of Education Shaanxi Normal University Xi'an China; ^5^ School of Agronomy and Horticulture Jiangsu Vocational College of Agriculture and Forestry Zhenjiang Jiangsu China; ^6^ College of Food Science and Biotechnology Zhejiang Gongshang University Hangzhou Zhejiang China; ^7^ USDA, ARS, U.S. Horticultural Research Laboratory Fort Pierce Florida USA

**Keywords:** ethylene, H_2_O_2_, PbrAPX14, PbrIDD2, pear, S‐sulfenylation

## Abstract

Endogenous H_2_O_2_ participated in the ethylene‐dependent ripening and senescence process of horticultural fruit as a secondary messenger; however, the molecular mechanism beneath such a phenomenon has not been fully clarified until recently. By a conjoint analysis of metabolite, enzyme activities, gene expression profiles in AsA‐GSH cycle of ‘Kolar’ pear, *PbrAPX14* might act as a negative factor in the ethylene‐mediated H_2_O_2_ accumulation. PbrAPX14, located in cytosol, would reduce H_2_O_2_ in vitro and in vivo, inhibit ethylene production, and thus fruit ripening and senescence. After analysing the expression profiles of the differentially expressed transcription factors (TFs) followed by experimental validation, the nuclear PbrIDD2 could directly bind to the *cis*‐acting element (core motif: TTTGTCG) in *PbrAPX14* promoter, activate its expression and thus enhance the H_2_O_2_‐scavenging capacity of fruit/calli, which was associated with the mitigated ethylene evolution and fruit ripening and senescence. Further study explored that the H_2_O_2_‐mediated post‐translational S‐sulfenylation of Cys^48^ residue in PbrAPX14, which mitigated its function, existed in vitro and in vivo, and was upregulated by ethylene, facilitating endogenous H_2_O_2_ accumulation. Overall, our results implied that both transcriptional and post‐translational regulation of PbrAPX14, which were (in)directly under the control of ethylene, functioned in pear ripening and senescence process via regulating endogenous H_2_O_2_ level.

## 1 Introduction

The ripening and senescence of climacteric fruit, such as tomato and pear, is a genetically programmed and developmentally regulated process, where climacteric ethylene plays an important role (Alba et al. 2005; Jiang et al. 2024). Climacteric ethylene, which relies on System 2, is biosynthesized from methionine by the sequential actions of S‐adenosylmethionine (SAM) synthetase (SAMS), 1‐amino‐cyclopropane carboxylate (ACC) synthase (ACS) and ACC oxidase (ACO), with SAM and ACC as intermediates (Alba et al. 2005). Upon biosynthesis, ethylene could bind to ethylene receptors (ETRs), with the aid of cuprous ion (Cu^+^) (Liu et al. 2015). Such interaction causes the inactivation of constitutive triple response 1 (CTR1), which promotes the cleavage of the carboxyl end in ethylene insensitive 2 (EIN2) (Wen et al. 2012). Then, the released C‐terminal fragment of EIN2 translocates from the endoplasmic reticulum into the nucleus to activate ethylene insensitive 3/ein3‐like 1 (SlEIN3/SlEIL1), or to the cytoplasmic processing body to repress the translation of *EIN3‐binding F‐box protein 1/2* (*SlEBF1/2*) mRNAs (Huang et al. 2022). Finally, the accumulation of EIN3/EIL1 promotes the transcription of downstream ethylene response factors (ERFs) (Shan et al. 2022). So far, several genes involved in climacteric ethylene biosynthesis (*PbrACS1b*, *PbrACO54*, etc.) and their upstream activators (*PbrERF24*, *PbrERF114*, etc.) have been characterized from the pear genome (Hao et al. 2018; Wang et al. 2024).

The impact of ethylene on fruit ripening and senescence process is supposedly associated with the metabolism of endogenous reactive oxygen species (ROS) (Huan et al. 2016; Kumar et al. 2016; Masia 2010; Pilati et al. 2014; Wang, Wang, et al. 2018). ROS are defined as a series of reduced or oxidized oxygen molecules, including hydrogen peroxide (H_2_O_2_), hypochlorous acid (HOCl), singlet oxygen (^1^O_2_), hydroxyl radical (·OH) and superoxide radical (O_2_·^−^) (Wang, Li, et al. 2021). Due to its relatively high stability in the biological system, H_2_O_2_ occupies a vital place, and many other ROS could be converted into H_2_O_2_ via various pathways (Li et al. 2010; Smirnoff and Arnaud 2019). Endogenous H_2_O_2_ and ethylene accumulated during ‘Ailsa Craig’ tomato ripening, with a burst at breaker stage and breaker +3 days, respectively (Dong et al. 2013; Gao et al. 2021; Kumar et al. 2016); when compared with those in the wild‐type ‘Ailsa Craig’ tomato, endogenous H_2_O_2_ level and peak were inhibited/delayed in the ripening‐impaired mutants (*ripening inhibitor* [*rin*], *nonripening* [*nor*] and *never ripe* [*Nr*]) with the lower ethylene production (Kumar et al. 2016; Osorio et al. 2011). Similarly, ROS metabolism was altered in ‘Housui’ pear after exogenous 1‐methylcyclopropene (1‐MCP, an inhibitor of ethylene perception) and ethrel (the ethylene donor) treatments (Wang, Wang, et al. 2018).

Additionally, exogenous application of H_2_O_2_ or the substrates for its formation (glycolic acid and xanthine) promoted the ripening and senescence process of ‘Kyoho’ grape (Guo et al. 2020; Guo et al. 2019), ‘Ailsa Craig’ tomato (Zhou et al. 2025), and ‘Bartlett’ pear (Brennan and Frenkel 1977); on the other hand, ‘Bartlett’ and ‘Bosc’ fruit treated with α‐hydroxy‐2‐pyridinemethanesulfonate, which would inhibit glycolate oxidase activity and thus endogenous peroxides production, displayed an opposite phenotype (Brennan and Frenkel 1977). By considering the fact that it is a signalling molecule in plants (Matilla‐Vázquez and Matilla 2011), these outcomes implied that endogenous H_2_O_2_ might participate in the ethylene‐dependent ripening and senescence process as a secondary messenger (Guo et al. 2020).

According to the literary review, the mechanism of the role of endogenous H_2_O_2_ in fruit ripening and senescence process has been proven to be bifunctional. RNA‐sequencing analysis revealed that exogenous H_2_O_2_ treatment promoted the early ripening of ‘Kyoho’ grape by regulating the expression profiles of genes involved in oxidative stress, cell wall deacetylation, cell wall degradation and photosynthesis (Guo et al. 2020). Besides, endogenous H_2_O_2_ could post‐translationally control the function of the ripening‐related proteins. AlkB homologue 2 (SlALKBH2), an m^6^A demethylase, is of great importance for normal ripening of tomato (Zhou et al. 2025); endogenous H_2_O_2_ could oxidize SlALKBH2 to facilitate homodimer formation, elevate protein stability, and thus enhance its function towards target transcripts including the pivotal ripening gene, *DNA demethylase 2* (*SlDML2*) (Zhou et al. 2025).

Ascorbate‐glutathione (AsA‐GSH) cycle, existing in various subcellular organs of plant cell (chloroplast, cytosol, mitochondrion, peroxisome, etc.), has proven to be the main metabolic pathway for endogenous H_2_O_2_ scavenging (Jiang et al. 2022). The basic framework of this cycle is similar (Kuźniak et al. 2017; Li et al. 2010; Wang, Ma, et al. 2021): ascorbate peroxidase (APX) utilizes AsA to reduce H_2_O_2_ with the concomitant production of monodehydroascorbate (MDHA); then, MDHA is either spontaneously converted to dehydroascorbate (DHA) or reduced by monodehydroascorbate reductase (MDHAR) to AsA; DHA, a short‐lived chemical, is reduced by dehydroascorbate reductase (DHAR) to AsA, using GSH as an electron donor; finally, glutathione disulfide (GSSG) produced in this cycle is converted back into GSH by glutathione reductase (GR), at the expense of NAD(P)H. Overexpression of a thylakoidal APX from *Solanum lycopersicum* (SlAPX) (Duan et al. 2012) or a peroxisomal APX from *Puccinellia tenuiflora* (PtAPX) (Guan et al. 2015) suppressed endogenous H_2_O_2_ accumulation in plants, while knockdown of the cytosolic APX1/2 from *Oryza sativa* (*OsAPX1/2*) illustrated an opposite impact (Rosa et al. 2010). A total of 31 genes in the AsA‐GSH cycle were characterized from the pear genome, with an uneven non‐random chromosomal distribution (Wang, Ma, et al. 2021); of these, *DHAR2* from *Pyrus sinkiangensis* (*PbDHAR2*; gene ID: Pbr016672.1) was validated to participate in endogenous H_2_O_2_ scavenging (Qin et al. 2015).

The abovementioned outcomes suggest that AsA‐GSH cycle might function in the ethylene‐dependent ripening and senescence process of horticultural fruit via regulating endogenous H_2_O_2_ level. In this study, *P. sinkiangensis* cv. ‘Kolar’ pear, which is popular among consumers due to its excellent organoleptic property (Jia et al. 2018), was used as material. A combination of quality and physio‐biochemical assays and plant molecular biological technologies was applied to clarify the role of candidate gene (*PbrAPX14*) in H_2_O_2_ scavenging and then the ethylene‐mediated ripening and senescence process of pear. Afterwards, an upstream regulator of *PbrAPX14*, named PbrIDD2, was characterized from pear genome with the aid of transcriptome result. Based on the result of pCysMod database (Li et al. 2021) and the characteristics of its isoforms in *Arabidopsis* (AtAPX1/5/6) (Huang et al. 2019), we performed an experiment and uncovered that the H_2_O_2_‐mediated S‐sulfenylation of PbrAPX14, which would weaken its function, took place in vitro and in vivo, and was regulated by ethylene. Additionally, the function of H_2_O_2_ in ethylene metabolism was also determined in this study.

## 2 Materials and Methods

### 2.1 Plant Material

#### 2.1.1 Experiment I

‘Kolar’ pear were harvested from homogeneous trees in an experimental orchard in Alar, Xinjiang, and then transported to laboratory; afterwards, uniform and defect‐free fruits were selected, placed on the shelves for 12 h at 0°C to emanate field heat, and then randomly divided into two lots for H_2_O (control) and 1.0 μL L^−1^ 1‐MCP fumigation for 24 h. Subsequently, fruit was dried or ventilated, packed with plastic bags before 0°C storage (90%–95% relative humidity [RH]) for 180 days. Samples were taken every 60 days. Here, we used the same sample for both control and 1‐MCP‐treated fruit on day 0; this sample was taken before H_2_O (control) and 1.0 μL L^−1^ 1‐MCP fumigation. There were three biological replicates per treatment, with 120 fruits per replicate.

#### 2.1.2 Experiment II

‘Kolar’ pear, uniform and defect‐free, was divided into three treatments: (i) H_2_O dipping for 5 min (control), (ii) 0.5 mL L^−1^ ethrel dipping for 5 min and (iii) 1.0 μL L^−1^ 1‐MCP fumigation for 24 h. After treatments, fruits were dried or ventilated, packed with plastic bags and then preserved at 20°C for 20 days. There were three biological replicates per treatment, with 90 fruit per replicate.

#### 2.1.3 Experiment III

‘Kolar’ pear, uniform and defect‐free, was soaked in H_2_O (control) or 10 mmol L^−1^ H_2_O_2_ for 60 min before storage at 20°C for 20 days. There were three biological replicates per treatment, with 30 fruit per replicate.

For the sampling at each stage, the first layer of cortex (below the pericarp) from the eight fruit per replicate was quickly removed with a sharp parer, immersed in liquid N_2_, fractured to pieces and then preserved at −80°C for the analysis of enzyme activity, gene expression profile, etc.; on the other hand, the other eight fruit per replicate were used for the determination of ethylene evolution, firmness, etc.

### 2.2 Ethylene‐Related Metabolite Determination

#### 2.2.1 Ethylene Evolution

Five fruits were weighed before incubation in a 2‐L glass jar for 30 min at 20°C. Then, 5.0‐mL headspace gas was extracted using a gas‐tight syringe before the determination of ethylene production by a gas chromatograph (GC, HP 5890A, Hewlett Packard, Avondale, PA) (Jia et al. 2023). Ethylene was identified and quantified by comparison of retention time and peak area with the standard.

#### 2.2.2 ACC Content

ACC in the cortex tissue was extracted and then analysed as described by Lindo‐García et al. (2020). Briefly, 5.0 g tissue was homogenized with 4 mL of 5% (w/v) sulfosalicylic acid. After shaking at 4°C for 30 min followed by centrifugation at 8000 *g* for 10 min at 4°C, 1.5 mL of the supernatant was collected and then mixed with 10 mmol L^−1^ HgCl_2_ and NaOCl saturated with NaOH (2:1, v/v). ACC content was calculated by monitoring ethylene formation in the headspace by a GC. The result was displayed as nmol C_2_H_4_ kg^−1^ FW.

### 2.3 Quality Attribute Analysis

#### 2.3.1 Colour

A Minolta CR‐400 chromameter (Konica Minolta Sensing Inc., Osaka, Japan) was used for the analysis of colour in the pericarp tissue (Wang, Ma, et al. 2018).

#### 2.3.2 Chlorophyll Analysis

Chlorophyll a and b in the pericarp tissue were assayed based on the method of Kozukue and Friedman (2003).

#### 2.3.3 Total Soluble Solids (TSS) and Titratable Acid (TA) Assay

TSS and TA in the cortex tissue were measured by a Pocket Brix‐Acidity Meter (PAL‐BX/ACID12, ATAGO, Tokyo, Japan) (Wang, Ma, et al. 2018).

#### 2.3.4 Firmness, Protopectin and Water‐Soluble Pectin Assay

Firmness of the cortex was measured by a hand penetrometer (GY‐1, Zhejiang, China) (Xie et al. 2017).

Protopectin and water‐soluble pectin in the cortex tissue were measured based on the protocol as described by Zhang et al. (2021). Galacturonic acid was used as an external standard for quantification, and the result was displayed as g galacturonic acid kg^−1^ FW.

### 2.4 Endogenous ROS Measurement

H_2_O_2_ in the cortex tissue was measured following the manual in the assay kit (H_2_O_2_‐1‐Y, Suzhou Comin Biotechnology Co. Ltd., Suzhou, China). The result was illustrated as mg kg^−1^ FW.

O_2_‧^−^ was detected following the method of Niu et al. (2024). Briefly, the cortex tissue was homogenized with 0.05 mol L^−1^ phosphate buffer (pH 7.8) and 10 mmol L^−1^ hydroxylamine hydrochloride, and then centrifuged at 4000 *g* for 10 min at 4°C before collection of the supernatant. O_2_‧^−^ was assayed after addition of 17 mmol L^−1^ sulfanilamide and 7 mmol L^−1^ α‐naphthylamine, with the aid of a UV–Vis spectrophotometer (UV‐2450/2550, Shimadzu, Japan). The result was expressed as mg kg^−1^ FW.

‧OH in the cortex tissue was assayed following the instructions in the assay kit (ADS‐W‐KY013, Jiangsu Aidisheng Biological Technology Co. Ltd., Yancheng, China). The result was displayed as μg kg^−1^ FW.

### 2.5 Crude Protein Extraction and Enzyme Activity Assay

#### 2.5.1 Ethylene Biosynthesis‐Related Enzymes

ACS crude enzyme extract was obtained after homogenization of 10.0 g of the cortex tissue with the extraction buffer, which contained 200 mmol L^−1^ tricine buffer (pH 8.5), 10 mmol L^−1^ dithiothreitol (DTT), 20 μmol L^−1^ pyridoxal phosphate and 2% (w/v) polyvinylpyrrolidone (PVP). The homogenate was then centrifuged at 18,000 *g* for 20 min at 4°C. Subsequently, 2.5 mL of the supernatant was loaded into a Sephadex G‐25 column (PD 10, Pharmacia, Madrid, Spain), which was previously equilibrated with 5 mmol L^−1^ tricine buffer (pH 8.0), 1 mmol L^−1^ DTT and 2 μmol L^−1^ pyridoxal 5ʹphosphate. After elution with the same buffer, 1.5 mL of sample was incubated with 200 mmol L^−1^ tricine buffer (pH 8.0), 100 μmol L^−1^ SAM for 2 h at 25°C. The reaction was stopped after the addition of 100 mmol L^−1^ HgCl_2_. Then, 1 mL of the sample was collected and mixed with 100 μL NaOCl saturated with NaOH (2:1 v/v). ACS activity was calculated by monitoring ethylene formation in the headspace by a GC (Chiriboga et al. 2012), and the result was expressed as μmol C_2_H_4_ s^−1^ kg^−1^ protein.

ACO activity was assayed based on the protocol of Chiriboga et al. (2012). Briefly, 10.0 g of sample was homogenized with 10 mL of extraction buffer containing 0.1 mol L^−1^ pH 7.4 Tris‐HCl, 10% glycerol, 30 mmol L^−1^ Na‐AsA, 5 mmol L^−1^ DTT and 1% (w/v) PVP. The homogenate was then centrifuged at 16,000 *g* for 20 min at 4°C. Subsequently, 2.5 mL of the supernatant was loaded into a Sephadex G‐25 column (PD 10, Pharmacia, Madrid, Spain), which was previously equilibrated with 20 mmol L^−1^ Tris‐HCl buffer (pH 7.4), 10% glycerol, 3 mmol L^−1^ Na‐AsA and 1 mmol L^−1^ DTT. After elution with the same buffer, 0.5 mL of sample was mixed with 10 mol L^−1^ FeSO_4_, 3 mmol L^−1^ sodium bicarbonate and 50 mol L^−1^ ACC before airing and incubation at 25°C for 20 min. ACO activity was calculated by monitoring ethylene formation in the headspace by a GC (Chiriboga et al. 2012), and the result was expressed as μmol C_2_H_4_ s^−1^ kg^−1^ protein.

#### 2.5.2 Enzymes in AsA‐GSH Cycle

Extraction and activity assay of APX, MDHAR, DHAR and GR in the cortex tissue were performed according to the manuals in the assay kits (APX‐1‐W, MDHAR‐1‐W, DHAR‐1‐W and GR‐1‐W, respectively; Suzhou Comin Biotechnology Co. Ltd., Suzhou, China) (Wang, Ma, et al. 2021). One unit of APX activity was expressed as the mass of oxidizing 1 μmol AsA into DHA per kilogram of protein per second at 25°C, and the result was expressed as μmol AsA s^−1^ kg^−1^ protein. One unit of MDHAR activity was expressed as the mass of oxidizing 1 μmol NADH into NAD^+^ per kilogram of protein per second at 25°C, and the result was expressed as μmol NADH s^−1^ kg^−1^ protein. One unit of DHAR activity was expressed as the mass of catalysing the formation of 1 μmol AsA per kilogram of protein per second at 25°C, and the result was expressed as μmol AsA s^−1^ kg^−1^ protein. On the other hand, one unit of GR activity was expressed as the mass of oxidizing 1 μmol NADPH into NADP^+^ per kilogram of protein per second at pH 8.0 at 25°C, and the result was expressed as μmol NADPH s^−1^ kg^−1^ protein.

Protein concentration in the crude enzyme extract was determined using the bicinchoninic acid protein assay kit (A045‐4, Nanjing Jiancheng Bioengineering Institute, Nanjing, China).

### 2.6 Transcriptome and qRT‐PCR Analysis

Transcriptome analysis was conducted according to the method of Wu et al. (2024). Briefly, total RNA was extracted from pear cortex using the EASYspin plant RNA extraction kit (Biomed, China), and then treated with DNase (Takara Biotechnology Co. Ltd., Dalian, China) to remove residual DNA before evaluation of RNA integrity, concentration and purity. Subsequently, the RNA‐seq library was constructed and sequenced by Novogene Technology Co. (Beijing, China) on the Illumina NovaSeq 6000 platform. After quality assessment and data filtering, clean reads were mapped to the pear genome via HISAT2 software. *P. bretschneideri* Rehd. cv. ‘Dangshansuli’ pear genome (Wu et al. 2013) was used as reference (based on the previous study of our laboratory, *P. sinkiangensis* was derived from a hybridization between *P. bretschneideri* and *P. communis* (Wu et al. 2018)). Fragments per Kilobase Million (FPKM) was used to calculate gene expression level, and the differentially expressed transcription factors (TFs) were identified with the DESeq software, in accordance with the following criteria: fold change ≥ 2.0 and *p*adj < 0.05.

qRT‐PCR assay was carried out as described by Wang, Ma, et al. (2018). The gene‐specific primers were designed using Primer Premier 6.0 (Table S1). Total RNA in the cortex tissue was isolated using TRizol Reagents (Invitrogen, USA) followed by RNase‐free DNase treatment (Qiagen, USA). After the synthesis of first‐strand cDNA, the qRT‐PCR assay was performed using TaKaRa One Step SYBR PrimeScript RT‐PCR Kit (Perfect Real Time) (Takara Biotechnology Co. Ltd., Dalian, China) (Wang, Ma, et al. 2018). *Tubulin* (*PbrTub*) and *PbrActin* were used as the internal reference genes for pear fruit and calli (Chen et al. 2015), while *SlActin‐51* and *SlCAC* were used as the housekeeping genes for the gene‐overexpressing tomato fruit (Liu et al. 2020; Zhang et al. 2018). The relative gene expression was calculated based on the 2^−ΔΔCT^ method (Wang, Ma, et al. 2021).

### 2.7 Measurement of PbrAPX14 Activity In Vitro

The open reading frame (ORF) of *PbrAPX14* was amplified from ‘Kolar’ pear (Table S1), inserted into the pCold‐TF plasmid and then introduced into *E. coli* BL21 (DE3) to express the His‐tagged fusion protein according to the protocol of Jia et al. (2024).

Kinetic assay of PbrAPX14 activity with AsA was performed with 1 mmol L^−1^ H_2_O_2_ in 50 mmol L^−1^ pH 7.0 potassium phosphate buffer based on the protocol of Zhang et al. (2023). AsA concentration varied from 50 to 1600 μmol L^−1^, the reaction was initiated by the addition of PbrAPX14, and the activity was calculated by the rate of AsA disappearance at 290 nm (Wang, Ma, et al. 2021).

### 2.8 Subcellular Localization Assay

The ORFs of *PbrAPX14* and *PbrIDD2*, without the stop codons, were amplified from ‘Kolar’ pear (Table S1), and then inserted into the pBI221 vector with a GFP tag. Subsequently, the recombinant plasmid was transformed, together with the corresponding marker, into *Arabidopsis* protoplasts before the fluorescence signal detection by a TCS SP5 confocal microscope (Leica Microsystems, Wetzlar, Germany), with the aid of Mrs. Lanfei Yin (BioRun Biosciences Co. Ltd.). AtH2B‐mcherry (Jia et al. 2024) was used as a nuclear indicator for PbrIDD2.

### 2.9 Gene Function Validation In Vivo

#### 2.9.1 Transient Overexpression of Gene in Pear Fruit

*PbrACO54*, *PbrAPX14* and *PbrIDD2* ORFs were cloned from ‘Kolar’ pear (Table S1), inserted into the modified pCAMBIA1300 vector with a GFP tag (named as pCAMBIA1301 vector), and then transformed into *Agrobacterium tumefaciens* strain GV3101 before incubation at 28°C until OD_660_ = 1.0. Following resuspension of the bacterial strain in the infiltration buffer, 5.0 μL of the solution was slowly injected into the cortex tissue of the mature ‘Kolar’ pear (Zhang et al. 2024). After 5‐d storage at 20°C (when compared with 0°C, 20°C would facilitate the gene‐induced quality and physio‐biochemical alternations), the cortex tissue from each injection site was sampled. Fruit infiltrated with the empty vector was used as the control. There were three biological replicates per treatment, with eight fruit per replicate.

#### 2.9.2 Transient Silence of Gene in Pear Fruit

Approximately 200‐bp fragments of *PbrAPX14* and *PbrIDD2* ORFs were cloned from ‘Kolar’ pear, and then inserted into the pTRV2 vector (Table S1). The recombinant plasmid and pTRV1 were transformed into *A. tumefaciens* strain GV3101, respectively. Subsequently, the bacterial resuspensions containing the recombinant pTRV2 and pTRV1, respectively, were mixed in a 1:1 ratio before injection into the cortex tissue of the mature ‘Kolar’ pear (Zhang et al. 2024). After 5‐d storage at 20°C (when compared with 0°C, 20°C would facilitate the gene‐induced quality and physio‐biochemical alterations), the cortex tissue from each injection site was sampled. Fruit co‐injected with the empty pTRV2 vector and pTRV1 was used as the control. There were three biological replicates per treatment, with eight fruits per replicate.

#### 2.9.3 Transformation of Pear Calli

*PbrAPX14* and *PbrIDD2* ORFs were amplified (Table S1), and then transformed into pear calli, which were induced from *P. communis* cv. ‘Clapp's Favourite’ fruitlet (Jia et al. 2024). After screening on the hygromycin B‐containing Murashige and Skoog (MS) medium (carbon source: sucrose), the positive lines were identified at the RNA level by qRT‐PCR and protein level by Western blotting. Finally, the positive transgenic lines were transferred to the hygromycin B‐containing MS medium with mixed sugar (sucrose/sorbitol (1:1), 15 g L^−1^) as the carbon source to simulate the condition of fruit growing on the tree and then grown at 20°C (a suitable temperature for calli growth) (Jia et al. 2024). Calli transformed with the empty vector was used as the control.

#### 2.9.4 Transformation of Tomato Fruit

The ORFs of *PbrAPX14* and *PbrIDD2*, after cloned from ‘Kolar’ pear (Table S1), were transformed into *S. lycopersicum* cv. ‘MicroTom’ based on the protocol of Zhang et al. (2024). The positive transgenic lines were screened on 100 mg L^−1^ kanamycin‐containing medium, and then confirmed at RNA level by qRT‐PCR and protein level by Western blotting. All plants were grown in the greenhouse (light for 18 h at 25°C and dark for 6 h at 18°C, 60% RH). Tomato fruit at 44 days after full bloom (DAFB) from the control (wide‐type) and transgenic homozygous lines (T2 generation) were sampled for further assay.

### 2.10 DNA and Protein Interaction Assay

#### 2.10.1 Dual‐Luciferase (Dual‐LUC)

*PbrIDD2* ORF was amplified from the ‘Kolar’ pear (Table S1), and then introduced into the pSAK277 vector; on the other hand, *PbrAPX14* promoter fragments, which contained diverse numbers of the wide‐type PbrIDD2‐binding element (core motif: TTTGTCG; *PbrAPX14pro* and *PbrAPX14pro* ^*frag1*^) or the mutated element (TTTGTCG→AAAAAAA; *PbrAPX14pro* ^*mut*^; mutation of the IDD2‐binding element was performed base on previous studies (Sun et al. 2019; Sun et al. 2020)), were inserted into the pGreen 0800‐LUC vector (Table S1), producing various reporters. Subsequently, a mixture of *A. tumefaciens* containing pSAK277‐*PbrIDD2* vector and each reporter was infiltrated into *N. benthamiana* leave. LUC image was captured by a Chemiluminescence Imager (SH‐Compact523, SHST, China), while LUC and Renilla (REN) activities were determined using a Dual‐LUC reporter assay system (Promega Corp., Madison, Wisconsin, USA) (Zhang et al. 2024). The transformant containing the empty pSAK277 vector and each reporter was used as the control.

#### 2.10.2 Yeast One‐Hybrid (Y1H)

*PbrIDD2* ORF was introduced into the prey vector pGADT7 (AD) (Table S1); on the other hand, about 200‐bp fragments of *PbrAPX14* promoter, containing the wide‐type PbrIDD2‐binding element (core motif: TTTGTCG; *PbrAPX14pro*) or the mutated element (TTTGTCG→AAAAAAA; *PbrAPX14pro* ^*mut*^), were inserted into the bait vector pAbAi (Table S1). Matchmaker Gold Yeast One‐Hybrid Library Screening System (Shanghai Weidi Industrial Co. Ltd., Shanghai, China) was applied to perform the Y1H assay (Jia et al. 2024). SD/‐Ura medium supplemented with AbA was used to check the self‐activation of *PbrAPX14pro* and *PbrAPX14pro* ^*mut*^, and then the appropriate AbA concentration was selected. Yeast cell co‐transformed with AD‐*p53* and *p53*‐AbAi was used as the positive control, while yeast co‐transformed with the empty AD vector and each bait as the negative control.

#### 2.10.3 Chromatin Immunoprecipitation qPCR (ChIP‐qPCR)

The *PbrIDD2*‐overexpressing calli and the control (empty vector) were used for the DNA‐protein cross‐link. After homogenization and cell lysis, chromatin was obtained, and then sonicated to get soluble sheared chromatins, with an average DNA length of 200‐500 bp. One part served as input DNA, while the other was used for immunoprecipitation with anti‐GFP antibody (Ab290, Abcam) (Zhang et al. 2024). The enrichment of *PbrAPX14* promoter fragment was evaluated via qPCR assay (Table S1).

#### 2.10.4 Electrophoretic Mobility Shift Assay (EMSA)

The His‐tagged recombinant PbrIDD2 protein was obtained based on the method as described in Section 2.7 (Table S1) (Jia et al. 2024). About 30‐bp biotin‐labelled DNA probes containing either wild‐type (core motif: TTTGTCG; *PbrAPX14pro*) or the mutated (TTTGTCG→AAAAAAA; *PbrAPX14pro* ^*mut*^) PbrIDD2‐binding site, and the unlabeled competitor probes, were synthesized by Sangong Bioengineering (Shanghai) Co. Ltd. EMSA assay was conducted based on the manual provided by the Chemiluminescent EMSA Kit (Beyotime Inc., Shanghai, China) (Jia et al. 2024).

### 2.11 Impact of Exogenous H_2_O_2_ Treatment on the Activities of PbrAPX14 and Its Mutants

To introduce Cys48Ser (substitution of cysteine residue at position 48 (Cys^48^) in PbrAPX14 with serine (Ser) residue, named as PbrAPX14^Cys48Ser^) and Cys20Ser and Cys78Phe and Cys184Ala (substitution of cysteine residues at positions 20, 78 and 184 with Ser, phenylalanine (Phe) and alanine (Ala) residues, respectively, named as PbrAPX14^Cys20Ser & Cys78Phe & Cys184Ala^) mutations into PbrAPX14, the pCold‐TF::PbrAPX14 plasmid was used as a template, and the mutation was achieved by overlapping PCR (Table S1). After confirmation of the mutation by DNA sequencing, the constructed plasmid was transformed into *E. coli* BL21 (DE3) for protein expression and then purification (Jia et al. 2024).

The impact of exogenous H_2_O_2_ supplementation on the activities of PbrAPX14 and its mutants was conducted based on the method of Hiner et al. (2000). Briefly, the recombinant protein was incubated with(out) 3 μmol L^−1^ H_2_O_2_; after removal of the excess H_2_O_2_ by the Micro Bio‐Spin P‐6 gel column (BioRad), the residual activity was assayed by APX assay kit (APX‐1‐W; Suzhou Comin Biotechnology Co. Ltd., Suzhou, China) (Wang, Ma, et al. 2021).

### 2.12 Analyses of the S‐Sulfenylated PbrAPX14 and Its Mutants After Exogenous H_2_O_2_ Treatment In Vitro

In brief, PbrAPX14 and its mutants (PbrAPX14^Cys48Ser^ and PbrAPX14^Cys20Ser & Cys78Phe & Cys184Ala^) were reduced by 1 mmol L^−1^ DTT for 1 h. After removal of the excess DTT by the Micro Bio‐Spin P‐6 gel column (BioRad), protein was incubated for 30 min at 20°C with 1 mmol L^−1^ dimedone, in the presence or absence of H_2_O_2_ (Waszczak et al. 2014). PbrAPX14‐SOH formation was analysed by immunoblot with an anti‐sulfenic acid modified cysteine (2‐thiodimedone‐specific Ig) antibody (Millipore).

### 2.13 Detection of the S‐Sulfenylated PbrAPX14 In Vivo

Due to its high similarity with other PbrAPXs, it is different to buy or produce the PbrAPX14‐specific antibody, which does not cross‐react with other isoforms and thus compromise the experimental accuracy. Thus, the *PbrAPX14*‐overexpressing ‘Kolar’ pear or ‘Clapp's Favourite’ fruitlet calli was used as material for this experiment (here, we used the modified pCAMBIA1300 vector with 3× Flag tag, named as pCAMBIA1302 vector).

After H_2_O_2_ and ethrel treatments, cortex tissue or calli was sampled, homogenized with lysis buffer and then centrifuged at 50,000 *g* for 10 min before incubation of the supernatant with 1 mmol L^−1^ DCP‐Bio1 probe (Millipore Corp., Massachusetts, USA) for 2 h. After treatment with the high‐capacity streptavidin‐agarose beads (Thermo Fisher Scientific Inc., Massachusetts, USA), the sample was subjected to a series of stringent wash, using 1% sodium dodecyl sulfate (SDS), 4 mol L^−1^ urea, 1 mol L^−1^ NaCl, 10 mmol L^−1^ DTT, 100 μmol L^−1^ ammonium bicarbonate and ultrapure water, before 10‐min boil. The S‐sulfenylated PbrAPX14 in vivo was detected by the immunoblot with anti‐3× Flag antibody (Ab290, Abcam) (Zhang et al. 2024).

For quantification of protein abundance, Image J software was used and signals from three independent experiments were quantified. Relative abundance of the S‐sulfenylated PbrAPX14 in vivo was calculated as the ratio of the S‐sulfenylated PbrAPX14‐Flag and loading control (PbrAPX14‐Flag) in the sample.

### 2.14 In Silico Analysis

Sequence alignment was performed using the DNAMAN software (Lynnon Biosoft, San Ramon, California, USA). A phylogenetic tree was constructed by MEGA7.0 software, using the maximum likelihood (ML) method, with a bootstrap analysis of 1000 replicates (Ma et al. 2020). Signal peptide was predicted by SignalP 5.0 database (https://services.healthtech.dtu.dk/services/SignalP‐5.0/), while transmembrane helix was characterized using TMHMM 2.0 database (https://services.healthtech.dtu.dk/services/TMHMM‐2.0/) (Carreón‐Anguiano et al. 2023). The PbrIDD2‐binding element in *PbrAPX14* promoter was analysed by PlantRegMap database (http://plantregmap.gao‐lab.org/) (Tian et al. 2020). On the other hand, the possible S‐sulfenylated cysteine (Cys) residue in PbrAPX14 was predicted via the pCysMod database (http://pcysmod.omicsbio.info/webserver.php) (Li et al. 2021).

### 2.15 Statistical Analysis

Data presented the mean value of three biological replicates. SAS version 9.3 (SAS Institute, Cary, NC) was used for the data analysis, in particular, analysis of variance (PROC ANOVA) with multi‐comparison correction. Mean separation was determined by Duncan's multiple range test at the 0.001, 0.01 and 0.05 levels. Spearman correlation coefficient between attributes was calculated by R software: extreme strong correlation was in the range of 0.8–1.0, and strong correlation was in the range of 0.6–0.8 (Long et al. 2014).

## 3 Results

### 3.1 Quality Alternation in ‘Kolar’ Pear After Exogenous 1‐MCP/Ethrel Treatment or Transient Overexpression of *PbrACO54* Gene

**FIGURE 1:**
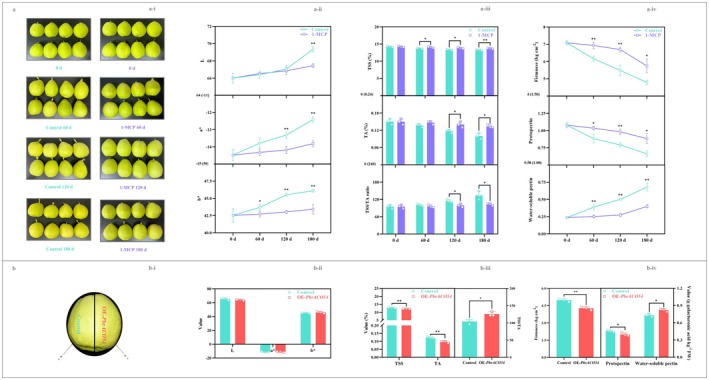
Quality alternation in ‘Kolar’ pear after exogenous 1‐MCP treatment or transient overexpression of *PbrACO54* gene. (a) Exogenous 1‐MCP treatment. (a‐i) Visual quality. (a‐ii) Colour. (a‐iii) TSS and TA. (a‐iv) Textural quality. ‘Kolar’ pears were treated with H_2_O (control) and 1‐MCP before storage at 0°C for 180 days; fruits were sampled every 60 days. (b) Transient overexpression of *PbrACO54* gene. (b‐i) Visual quality. (b‐ii) Colour. (b‐iii) TSS and TA. (b‐iv) Textural quality. ‘Kolar’ pear transformed with the empty pCAMBIA1301 vector was used as the control for the OE fruit. After infiltration, fruits were preserved at 20°C for 5 days before sampling. Data represent the mean value of three biological replicates, and significant difference (*p < 0.05; **p < 0.01; ***p < 0.001) between samples at the same sampling time is determined via one‐way ANOVA.

As shown in Figure 1a–i,ii, *L*, a* and b* accumulated with the prolonged 0°C storage of ‘Kolar’ pear; consistently, chlorophyll a and b contents decreased during storage (Figure S1). TSS and TA gradually reduced in association with the increment of TSS/TA ratio (Figure 1a–iii). For the textural quality, protopectin continuously decreased, while water‐soluble pectin increased, causing the reduction of fruit firmness (Figure 1a–iv).

Exogenous 1‐MCP fumigation suppressed colour transition, chlorophyll degradation and softening process, and maintained taste quality (TSS and TA) during 0°C storage of ‘Kolar’ pear (Figures 1a and S1); on the other hand, an opposite phenomenon was observed in the *PbrACO54*‐overexpressing fruit (Figure 1b; because we just injected the gene into the cortex tissue; therefore, no difference in colour was detected between two samples). A similar outcome was observed after 1‐MCP and ethrel treatments of ‘Kolar’ pear before 20°C storage (Figure S2a).

### 3.2 Alternation of Ethylene Metabolism in ‘Kolar’ Pear After Exogenous 1‐MCP/Ethrel Treatment or Transient Overexpression of *PbrACO54* Gene

**FIGURE 2:**
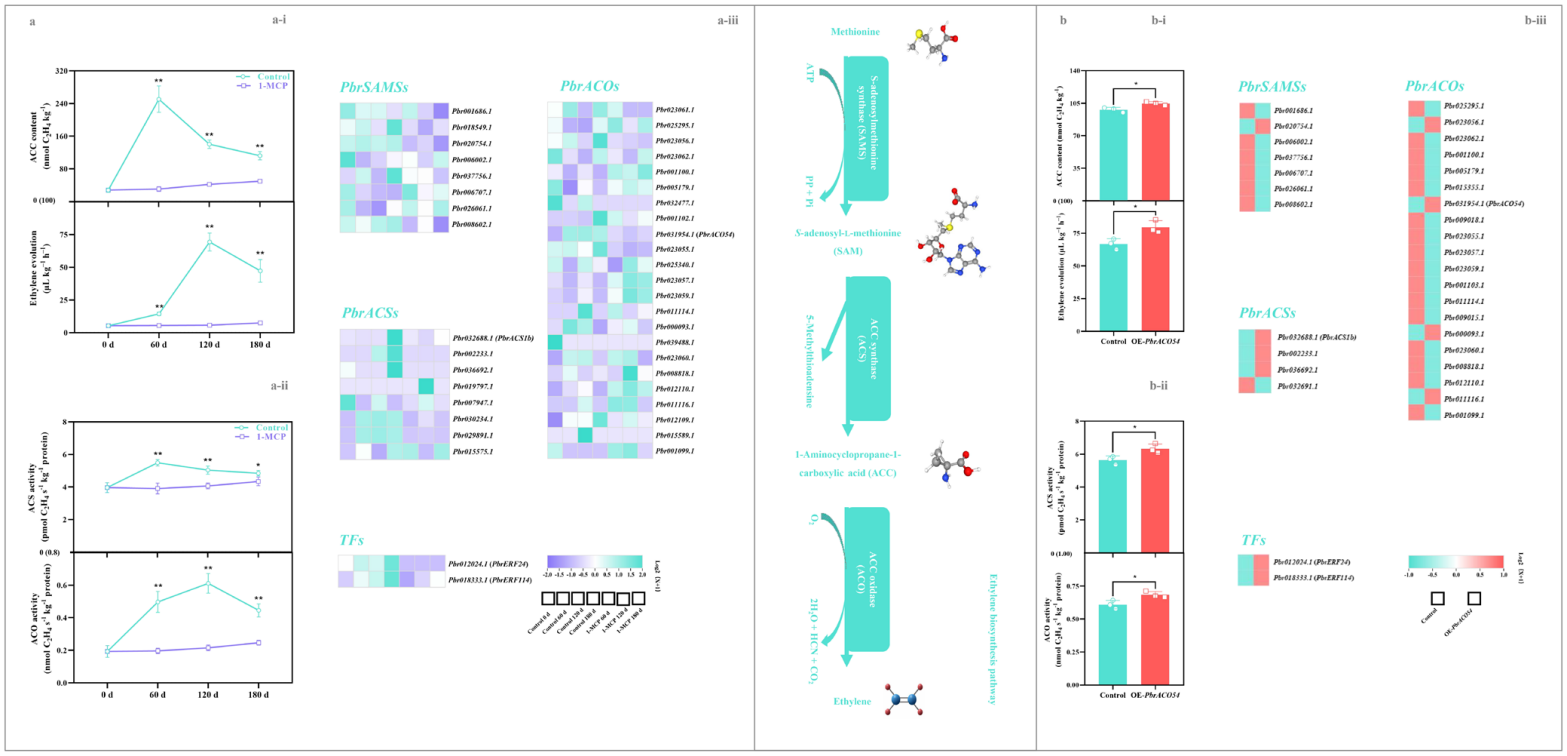
Alternation of ethylene metabolism in ‘Kolar’ pear after exogenous 1‐MCP treatment or transient overexpression of *PbrACO54* gene. (a) Exogenous 1‐MCP treatment. (a‐i) ACC and ethylene contents. (a‐ii) Enzyme activities. (a‐iii) Gene expression profiles (data were adapted from transcriptome assay). ‘Kolar’ pears were treated with H_2_O (control) and 1‐MCP before storage at 0°C for 180 days; fruits were sampled every 60 days. Colour scale represents normalized log_2_‐transformed (mean FPKM +1), where cyan indicates a high level, purple presents a low level and white demonstrates a medium level (genes, whose expression could not be detected at all samples, were not illustrated in the figure). (b) Transient overexpression of *PbrACO54* gene. (b‐i) ACC and ethylene contents. (b‐ii) Enzyme activities. (b‐iii) Gene expression profiles (data were adapted from transcriptome assay). ‘Kolar’ pear transformed with the empty pCAMBIA1301 vector was used as the control for the overexpressing (OE) fruit. After infiltration, fruits were preserved at 20°C for 5 days before sampling. Colour scale represents normalized log_2_‐transformed (mean FPKM +1), where red indicates a high level, cyan presents a low level and white demonstrates a medium level (genes, whose expression could not be detected at all samples, were not illustrated in the figure). Data represent the mean value of three biological replicates, and significant difference (*p < 0.05; **p < 0.01; ***p < 0.001) between samples at the same sampling time is determined via one‐way ANOVA.

As shown in Figure 2a–i, ethylene accumulated during 0°C storage of ‘Kolar’ pear, with a climacteric peak on the 120th day. Consistent with this, ACO and ACS activities as well as ACC content in its biosynthetic pathway increased, with a peak on the 60th day or 120th day (Figure 2a–i,ii). Based on transcriptome annotation, eight *PbrSAMSs*, 15 *PbrACSs* and 23 *PbrACOs* were expressed, with diverse expression patterns (Figure 2a–iii and Table S2). Because *PbrACS1b* and *PbrACO54* as well as their upstream regulators, *PbrERF24* and *PbrERF114*, were previously validated to participate in ethylene biosynthesis of the ripening pear (Hao et al. 2018; Wang et al. 2024), we then paid attention to these genes. As shown in Figure 2a–iii and Table S2, the mRNA abundances of *PbrACS1b*, *PbrERF24* and *PbrERF114* demonstrated an increasing trend with the prolonged storage time, while *PbrACO54* transcripts gradually accumulated with a peak at the 120th day and then declined.

Exogenous 1‐MCP fumigation considerably suppressed *PbrACS1b*, *PbrACO54*, *PbrERF24*, *PbrERF114* expression levels, ACO and ACS activities and ACC content throughout 0°C storage, causing the lower ethylene production than that in control fruit (Figure 2a and Table S2). On the other hand, an opposite phenomenon was observed in the *PbrACO54*‐overexpressing ‘Kolar’ pear with the elevated ethylene evolution (Figure 2b and Table S3). qRT‐PCR validated transcriptome results on the expression profiles of the abovementioned four genes (Figure S3a–i,b–i and Tables S2 and S3). Similar outcome was observed after 1‐MCP and ethrel treatments of ‘Kolar’ pear before 20°C storage (Figure S2b).

In combination of the outcomes in Figures 1, 2, S1 and S2a,b, the alternation of colour, chlorophyll a and b, TSS and TA and firmness in the ripening ‘Kolar’ pear was regulated by ethylene.

### 3.3 Characterization of *PbrAPX14* as the Candidate Gene Responsible for Endogenous H_2_O_2_ Scavenging

**FIGURE 3:**
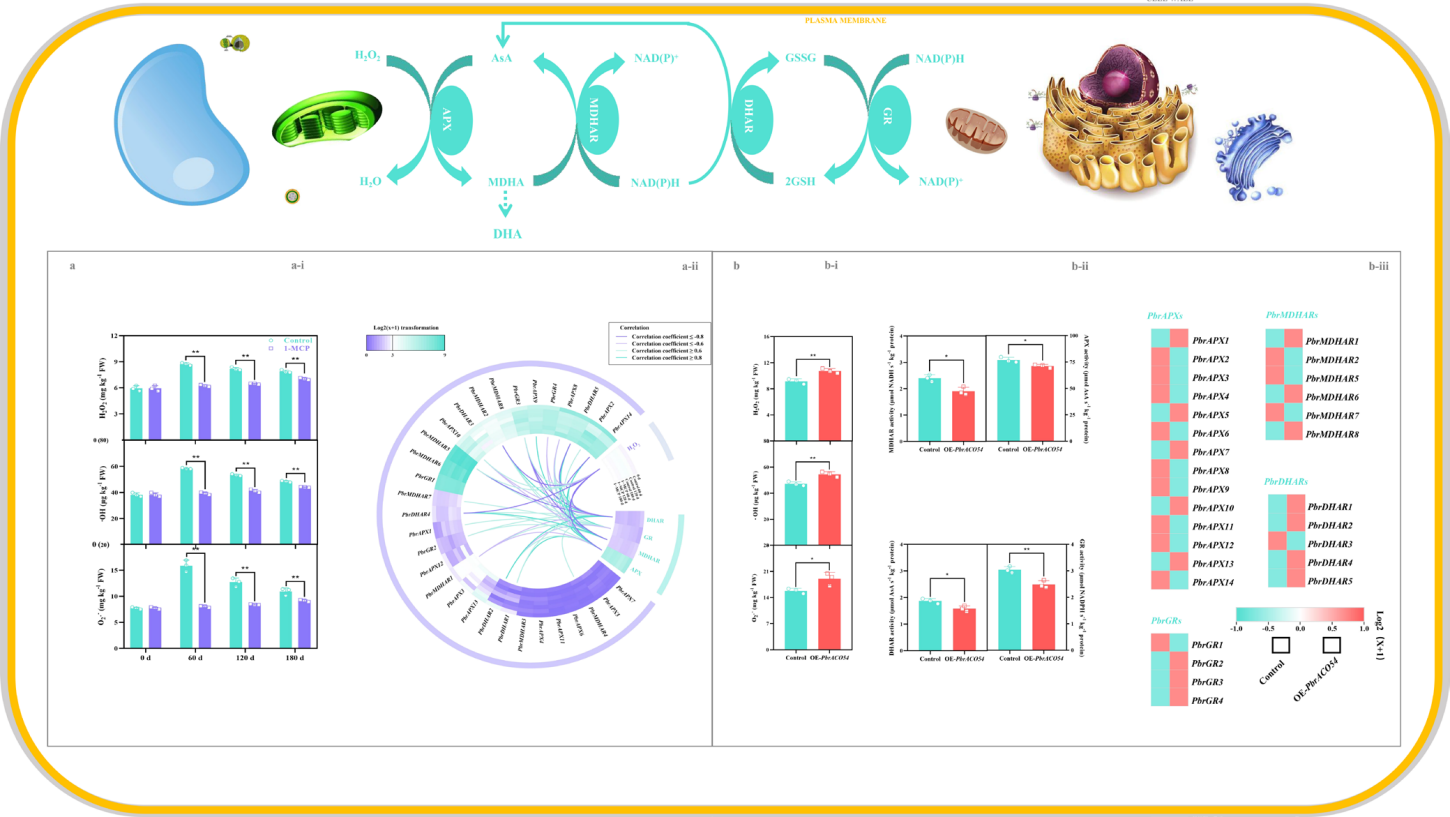
Characterization of *PbrAPX14* as the candidate gene responsible for the alternation of H_2_O_2_. (a) Impact of exogenous 1‐MCP treatment on the metabolism of AsA‐GSH cycle. (a‐i) Endogenous ROS. (a‐ii) Enzyme activities and gene expression profiles (data were adapted from transcriptome assay) as well as their correlations with H_2_O_2_ level. ‘Kolar’ pears were treated with H_2_O (control) and 1‐MCP before storage at 0°C for 180 days; fruits were sampled every 60 days. Colour scale represents normalized log_2_‐transformed (mean value +1), where cyan indicates a high level, purple presents a low level and white demonstrates a medium level. Spearman correlation between different attributes is visualized in the heatmap, where cyan (or light cyan) line demonstrates extreme strong (or strong) positive correlation, while purple (or light purple) line indicates extreme strong (or strong) negative association. (b) Impact of transient overexpression of *PbrACO54* gene on the metabolism of AsA‐GSH cycle. (b‐i) Endogenous ROS. (b‐ii) Enzyme activities. (b‐iii) Gene expression profiles (data were adapted from transcriptome assay). ‘Kolar’ pear transformed with the empty pCAMBIA1301 vector was used as the control for the OE fruit. After infiltration, fruits were preserved at 20°C for 5 days before sampling. Colour scale represents normalized log_2_‐transformed (mean value +1), where red indicates a high level, cyan presents a low level and white demonstrates a medium level (genes, whose expression could not be detected at all samples, were not illustrated in the figure). Data represent the mean value of three biological replicates, and significant difference (*p < 0.05; **p < 0.01; ***p < 0.001) between samples at the same sampling time is determined via one‐way ANOVA.

Endogenous ROS, including H_2_O_2_, ‧OH and O_2_·^−^, accumulated during the early storage period and then declined, with a peak at 60th day (Figure 3a–i). Exogenous 1‐MCP fumigation suppressed ROS accumulation (Figure 3a–ii); on the other hand, an opposite phenomenon was observed in the *PbrACO54*‐overexpressing fruit (Figure 3b–i).

H_2_O_2_, which has been validated to participate in fruit ripening and senescence process (Kumar et al. 2016; Zhou et al. 2025), is mainly scavenged by AsA‐GSH cycle (Jiang et al. 2022). Subsequently, we intended to the candidate genes responsible for the alternation of endogenous H_2_O_2_ in postharvest ‘Kolar’ pear. As shown in Figure 3a–ii, GR activity steadily increased, while APX, DHAR and MDHAR activities demonstrated a decreasing trend with the prolonged storage time at 0°C. The activities of all four enzymes were upregulated by exogenous 1‐MCP treatment, but suppressed after transient overexpression of *PbrACO54* (Figure 3a–ii,b–ii). Based on transcriptome results, all 31 genes in AsA‐GSH cycle (Wang, Ma, et al. 2021) were transcribed during 0°C storage of ‘Kolar’ pear, with diverse expression patterns (Figure 3a–ii and Table S4). Of these, only *PbrAPX14*, whose expression was upregulated by 1‐MCP treatment, but suppressed after transient overexpression of *PbrACO54*, displayed extremely strong positive correlations with the corresponding enzyme (APX) activity, but extremely strong negative correlations with H_2_O_2_ (absolute correlation coefficient ≥ 0.8; Figure 3a–ii,b–ii,iii; Tables S4 and S5). qRT‐PCR validated transcriptome results on *PbrAPX14* expression profiles (Figure S3a–ii,b–ii). Similar outcome was observed after 1‐MCP/ethrel treatment of ‘Kolar’ pear before 20°C storage (Figure S2c–f).

Taken together, these results implied that the metabolism of endogenous ROS (H_2_O_2_, ‧OH and O_2_·^−^) was under the control of ethylene; and *PbrAPX14* might be the candidate gene involved in endogenous H_2_O_2_ scavenging and thus the ethylene‐mediated ripening and senescence process of ‘Kolar’ pear. Thus, it was selected for further study.

### 3.4 Functional Validation of *PbrAPX14* In Vitro and In Vivo

As shown in Figure S4a and Table S6, PbrAPX14 protein sequence was highly identical with the cytosolic APXs from other plants, possessing the same AsA‐binding residues (Chin et al. 2019). Further study uncovered that PbrAPX14 catalyses the oxidation of AsA into DHA, with *K*_*m*_ and *V*_max_ of 461.30 μM and 352.52 s^−1^, respectively (Figure S4b).

**FIGURE 4:**
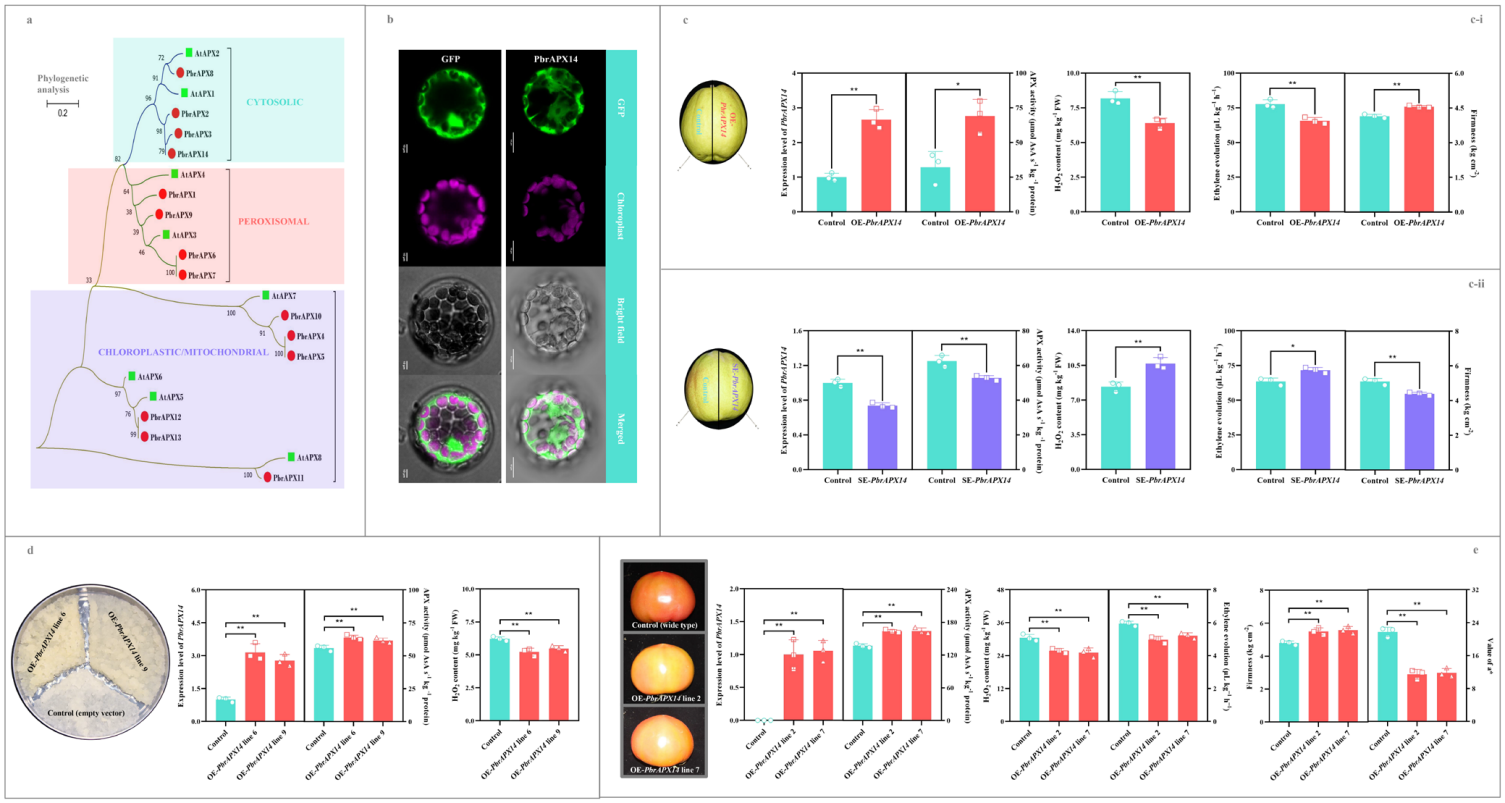
Functional validation of *PbrAPX14* gene in vivo. (a) Phylogenetic analysis of APXs from pear and *Arabidopsis*. Information on APXs from pear and *Arabidopsis* was summarized in Table S6. (b) Subcellular localization of PbrAPX14. (c) Functional validation of *PbrAPX14* gene in pear fruit. (c‐i) Transient overexpression of *PbrAPX14*. ‘Kolar’ pear transformed with the empty pCAMBIA1301 vector was used as the control for the OE fruit. (c‐ii) Transient silence of *PbrAPX14*. Fruit co‐transformed with the empty pTRV2 and pTRV1 was used as the control for the silenced (SE) pear. After infiltration, fruits were preserved at 20°C for 5 days before sampling. The expression level of *PbrAPX14* in the control fruit was set as 1.0 for qRT‐PCR. (d) Functional validation of *PbrAPX14* gene in pear calli. Calli transformed with the empty pCAMBIA1301 vector was used as the control for the OE calli. After transformation, calli was grown at 20°C. The expression level of *PbrAPX14* in the control calli was set as 1.0 for qRT‐PCR. (e) Functional validation of *PbrAPX14* gene in tomato fruit. The wide‐type tomato was used as the control for the OE fruit. All plants were grown in the greenhouse (light for 18 h at 25°C and dark for 6 h at 18°C, 60% RH) before sampling tomato fruit at 44 DAFB. The expression level of *PbrAPX14* in the OE*‐PbrAPX14* line 2 was set as 1.0 for qRT‐PCR. Data represent the mean value of three biological replicates, and significant difference (*p < 0.05; **p < 0.01; ***p < 0.001) between samples is determined via one‐way ANOVA.

Subsequently, we detected the function of *PbrAPX14* in vivo. Consistent with the result of phylogenetic analysis, PbrAPX14, without any signal peptide and transmembrane domain, was located in cytosol (Figures 4a,b and S5a). Transient overexpression of *PbrAPX14* in the cortex tissue of ‘Korla’ pear, ‘Clapp's Favourite’ calli and ‘MicroTom’ tomato upregulated APX activity, and thus decreased endogenous H_2_O_2_ level, which was concomitant with the suppressed ethylene evolution, softening process and colour alternation in fruit (Figures 4c–e and S6a); on the other hand, a reverse phenomenon was observed in the *PbrAPX14*‐silenced ‘Kolar’ pear (Figure 4c–ii).

### 3.5 Identification and Validation of PbrIDD2 as the Upstream Activator of *PbrAPX14*

In horticultural fruit, TFs (in)directly regulated the transcription of the downstream structural genes (Jia et al. 2024). Therefore, we tried to identify the candidate TFs regulating *PbrAPX14* expression. Based on RNA‐sequencing result, 456 out of 559 differentially expressed TFs displayed continuous higher (or lower) mRNA abundances in the 1‐MCP‐treated fruit than those in the control throughout 0°C storage (Figure S7a and Table S7, fold change ≥ 2.0 and *p*adj < 0.05 for at least one stage); of these, 40 members, whose expression was upregulated by 1‐MCP fumigation, demonstrated extremely strong positive associations with *PbrAPX14* (correlation coefficient ≥ 0.8; Figure S7b and Table S7). Further analysis found that 18 out of the abovementioned 40 differentially expressed TFs displayed higher mRNA abundances in the *PbrACO54*‐overexpressing fruit than those in the control (Figure S7c and Table S8, fold change ≥ 2.0 and *p*adj < 0.05), implying that they might be the upstream activators of *PbrAPX14*. Because of its relatively high coefficient with *PbrAPX14* during ‘Kolar’ pear storage at 0°C (Figure S7b), *Pbr006167.1*, named as *PbrIDD2* by Su et al. (2019), was selected for further study. qRT‐PCR validated transcriptome results on *PbrIDD2* expression profiles (Figure S3a–ii,b–ii). A similar outcome was found after 1‐MCP and ethrel treatments of ‘Kolar’ pear before 20°C storage (Figure S2e,f).

**FIGURE 5:**
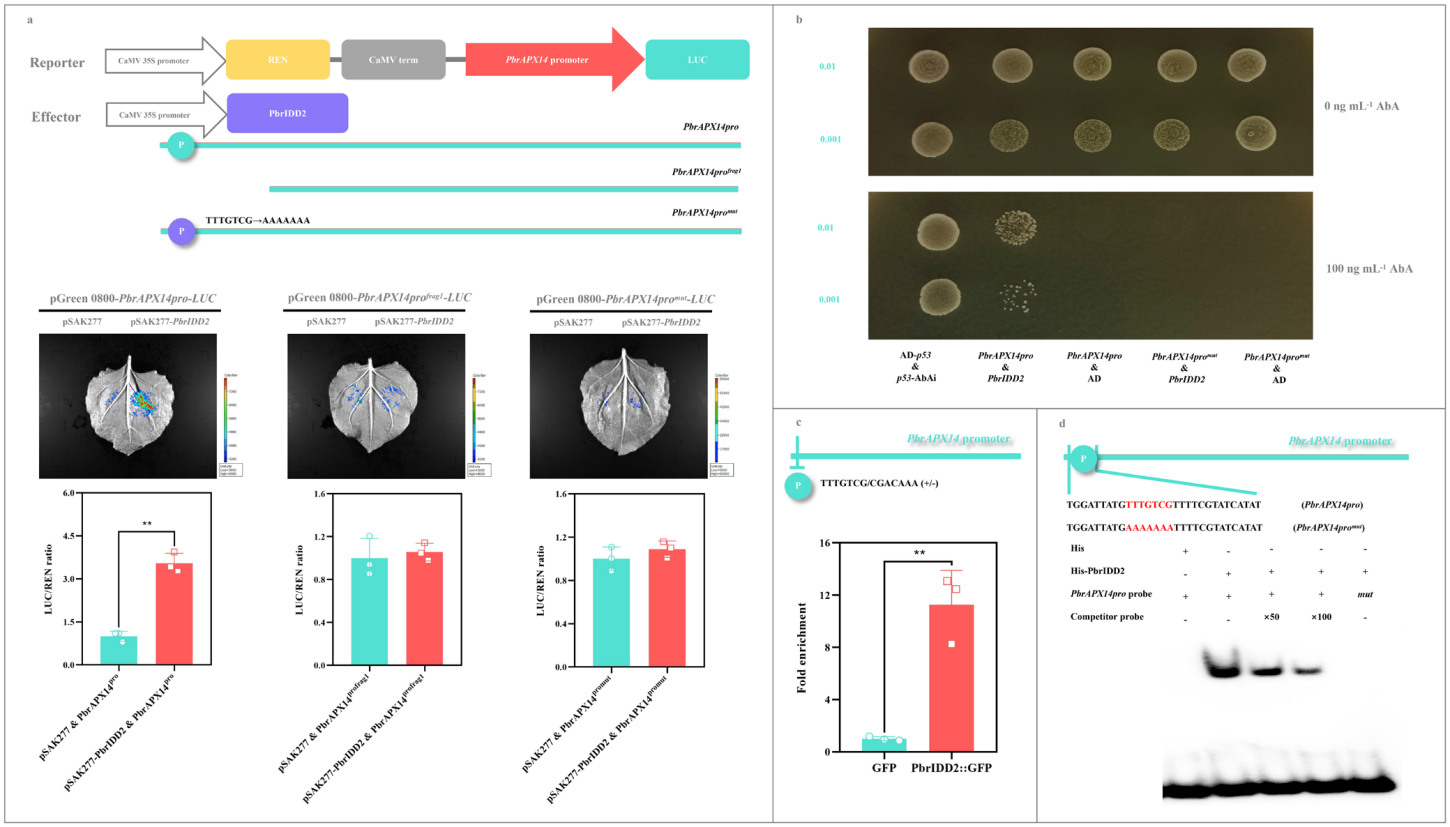
Confirmation of PbrIDD2 as the upstream activator of *PbrAPX14* gene. (a) Dual‐LUC assay. Transformant containing the empty pSAK277 vector and each reporter was used as the control. (b) Y1H measurement. Yeast cell co‐transformed with AD‐*p53* and *p53*AbAi was used as the positive control, while yeast co‐transformed with the empty AD vector and each bait as the negative control. (c) ChIP‐PCR analysis. Calli overexpressing the empty vector was used as a negative control. (d) EMSA text. The biotin‐labelled *PbrAPX14* promoter fragments containing the PbrIDD2‐binding element (core motif: TTTGTCG) and its mutant (TTTGTCG→AAAAAAA) were named as *PbrAPX14pro* probe and *PbrAPX14pro* ^*mut*^ probe, respectively; and the unlabeled *PbrAPX14* promoter fragment containing the PbrIDD2‐binding element was used as competitor probe. The presence and absence of His protein, His‐PbrIDD2 protein, biotin‐labelled probe, or competitor probe were indicated by ‘+’ and ‘−’, respectively. Competitor probe concentrations were 50‐fold (50×) and 100‐fold (100×) those of the labelled probe. Data represent the mean value of three biological replicates, and significant difference (*p < 0.05; **p < 0.01; ***p < 0.001) between samples is determined via one‐way ANOVA.

Based on the result of the PlantRegMap database (Tian et al. 2020), PbrIDD2 might interact with one *cis*‐acting element (core motif: TTTGTCG) in *PbrAPX14* promoter (Figure S7d). Then, we tried to validate such hypothesis. As shown in Figure 5a, about 3.5‐fold increase in LUC/REN ratio was observed in tobacco leave co‐transformed with *PbrIDD2* and reporter containing *PbrAPX14* promoter; and such increment was disappeared after the cleavage or mutation of the PbrIDD2‐binding element (Figure 5a). In the Y1H assay, the positive control (AD‐*p53* and *p53*AbAi), negative control (AD and *PbrAPX14pro*‐pAbAi), bait‐prey co‐transformant (AD‐*PbrIDD2* and *PbrAPX14pro*‐pAbAi) and co‐transformants containing the mutated *cis*‐acting element (AD and *PbrAPX14pro* ^*mut*^‐pAbAi, AD‐*PbrIDD2* and *PbrAPX14pro* ^*mut*^‐pAbAi) grew normally on SD/−Leu medium (Figure 5b); when AbA was added, the growth of the negative control as well as transformants possessing the mutated binding element was inhibited, without any influence on the positive control and bait‐prey co‐transformant (Figure 5b). ChIP‐qPCR analysis caused about 11‐fold enrichment in *PbrAPX14* promoter fragment harbouring the PbrIDD2‐binding element in comparison with the control (Figure 5c). In vitro EMSA experiment found that the protein‐DNA complex was formed when His‐PbrIDD2 was incubated with the labelled probe, and such binding was gradually inhibited with the increased abundance of the unlabeled competitor probe (Figure 5d); on the other hand, the complex disappeared after the mutation of the binding element (Figure 5d).

In combination, our results implied that PbrIDD2 could interact with one *cis*‐acting element (core motif: TTTGTCG) in *PbrAPX14* promoter and then activate its expression.

### 3.6 Functional Validation of PbrIDD2 In Vivo

**FIGURE 6:**
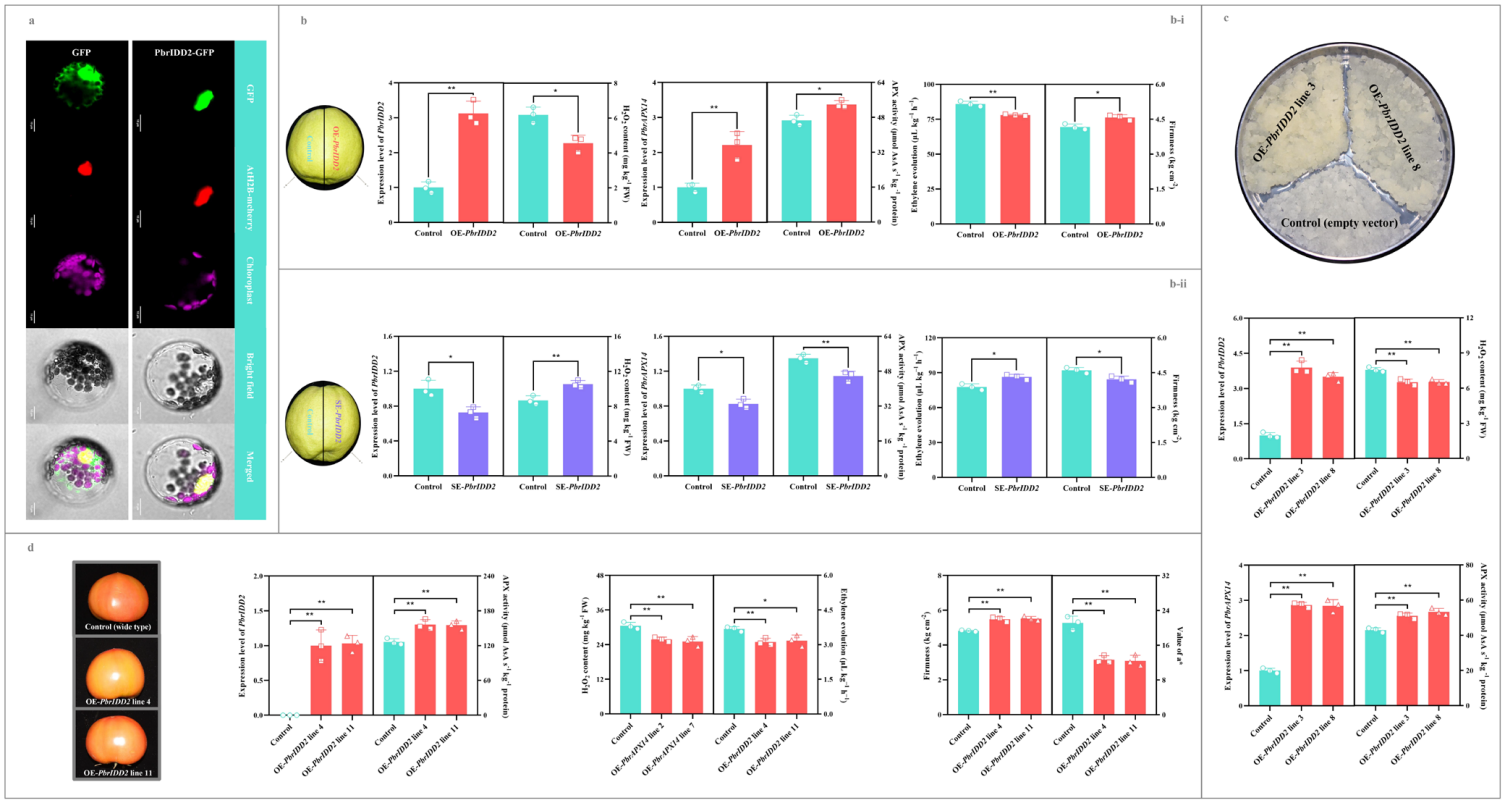
Functional validation of *PbrIDD2* gene. (a) Subcellular localization of PbrIDD2. AtH2B‐mcherry (Jia et al. 2024) was used as a nuclear indicator. (b) Functional validation of *PbrIDD2* gene in pear fruit. (b‐i) Transient overexpression of *PbrIDD2*. ‘Kolar’ pear transformed with the empty pCAMBIA1301 vector was used as the control for the OE fruit. (b‐ii) Transient silence of *PbrIDD2*. Fruit co‐transformed with the empty pTRV2 and pTRV1 was used as the control for the SE pear. After infiltration, fruits were preserved at 20°C for 5 days before sampling. The expression level of each gene in the control fruit was set as 1.0 for qRT‐PCR. (c) Functional validation of *PbrIDD2* gene in pear calli. Calli transformed with the empty pCAMBIA1301 vector was used as the control for the OE calli. After transformation, calli were grown at 20°C. The expression level of each gene in the control calli was set as 1.0 for qRT‐PCR. (d) Functional validation of *PbrIDD2* gene in tomato fruit. The wide‐type tomato was used as the control for the OE fruit. All plants were grown in the greenhouse (light for 18 h at 25°C and dark for 6 h at 18°C, 60% RH) before sampling tomato fruit at 44 DAFB. The expression level of *PbrIDD2* in the OE‐*PbrIDD2* line 4 was set as 1.0 for qRT‐PCR. Data represent the mean value of three biological replicates, and significant difference (*p < 0.05; **p < 0.01; ***p < 0.001) between samples is determined via one‐way ANOVA.

Subsequently, we made an effort to validate the role of PbrIDD2 in vivo. As shown in Figures 6a and S5b, PbrIDD2, without any signal peptide and transmembrane domain, accumulated in the nucleus of *Arabidopsis* protoplast.

Transient overexpression of *PbrIDD2* in ‘Korla’ pear, ‘Clapp's Favourite’ calli and ‘MicroTom’ tomato substantially upregulated *PbrAPX14* mRNA abundance and APX activity, resulting in lower endogenous H_2_O_2_ content than that in the controls (Figures 6b–d and S6b); additionally, ethylene evolution, softening process and colour transition in the transgenic fruits were inhibited as well (Figure 6b,d). On the other hand, a reverse phenomenon was observed in the *PbrIDD2*‐silenced ‘Kolar’ pear (Figure 6b–ii).

### 3.7 Detection of the S‐Sulfenylated PbrAPX14 In Vitro and In Vivo

**FIGURE 7:**
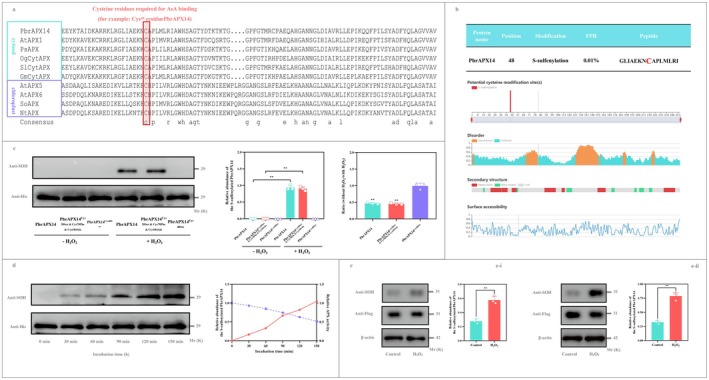
Detection of the S‐sulfenylated PbrAPX14 in vitro and in vivo. (a) Alignment of the cytosolic and chloroplastic APXs in plants. Information on the cytosolic and chloroplastic APXs in plants was summarized in Table S6. The conserved cysteine residues responsible for AsA‐binding were highlighted in the box based on the outcomes of previous studies (Kitajima 2008; Kitajima et al. 2008; Chin et al. 2019); moreover, abovementioned sites in AtAPX1 and AtAPX5/6 could be S‐sulfenylated by H_2_O_2_ (Huang et al. 2019). Just part of the result was illustrated. (b) Prediction of the possible S‐sulfenylated cysteine residue in PbrAPX14. The possible S‐sulfenylated cysteine residue was predicted by pCysMod database (Li et al. 2021). (c) Experimental validation of the possible S‐sulfenylated cysteine residue in PbrAPX14 in vitro. APX activity in each protein (PbrAPX14, PbrAPX14^Cys48Ser^ and PbrAPX14^Cys20Ser & Cys78Phe & Cys184Ala^) without H_2_O_2_ treatment was set as 1.0. (d) Impact of exogenous H_2_O_2_ incubation time on the abundance of the S‐sulfenylated PbrAPX14 and APX activity. The residual activity was expressed as a percentage of the initial (0 min). (e) Impact of exogenous H_2_O_2_ treatment on the abundance of the S‐sulfenylated PbrAPX14 in vivo. (e‐i) Pear fruit. After transformation with *PbrAPX14*, ‘Kolar’ pears were divided into two groups for H_2_O (control) and 10 mmol L^−1^ H_2_O_2_ treatments before sampling after 20°C storage for 5 days. (e‐ii) Pear calli. *PbrAPX14*‐overexpressing ‘Clapp's Favourite’ fruitlet calli were treated with H_2_O (control) and or 2 mmol L^−1^ H_2_O_2_ before incubation at 20°C for 20 days. Relative abundance of the S‐sulfenylated PbrAPX14 in vivo was calculated as the ratio of the S‐sulfenylated PbrAPX14‐Flag and loading control (PbrAPX14‐Flag). Data represent the mean value of three biological replicates, and significant difference (*p < 0.05; **p < 0.01; ***p < 0.001) between samples is determined via one‐way ANOVA.

As shown in Figure S8, about 60% reduction of APX activity was detected after mutation of the Cys^48^ residue in PbrAPX14, which was conserved in plant cytosolic/chloroplastic APXs for AsA binding (Figure 7a and Table S6) (Kitajima 2008; Kitajima et al. 2008; Chin et al. 2019). Similar phenomena were observed after mutation of the corresponding cysteine residue in a cytosolic APX1 from *Arabidopsis* (AtAPX1) (Yang et al. 2015) or a chloroplastic APX from *Nicotiana tabacum* (NtAPX) (Kitajima et al. 2008) (Figure 7a and Table S6).

Thiol group on cysteine might undergo multiple post‐translational modifications, altering protein characteristics (Huang et al. 2019; Li et al. 2021). With the aid of pCysMod database (Li et al. 2021), only Cys^48^ residue in PbrAPX14 (also the AsA‐binding residue) might be S‐sulfenylated by H_2_O_2_ (FPR < 5%; Figure 7b); moreover, the corresponding residues in the cytosolic AtAPX1 (Cys^32^ residue) and chloroplastic AtAPX5/6 (Cys^102^ and Cys^123^ residues, respectively) could be S‐sulfenylated by H_2_O_2_ based on the result of LC‐MS/MS analysis (Figure 7a and Table S6) (Huang et al. 2019). Therefore, a further experiment was conducted to validate this hypothesis.

We first obtained the wide‐type PbrAPX14 and its mutants (PbrAPX14^Cys48Ser^ and PbrAPX14^Cys20Ser & Cys78Phe & Cys184Ala^; here, we mutated cysteine residues with other residues based on the report of Yang et al. (2015) and alignment outcome in Figure S4a), and then treated with H_2_O_2_. As shown in Figure 7c, no S‐sulfenylated protein and thus enzyme activity change was detected after incubation of PbrAPX14^Cys48Ser^ with H_2_O_2_; on the other hand, an opposite phenomenon was detected for PbrAPX14 and PbrAPX14^Cys20Ser & Cys78Phe & Cys184Ala^: the S‐sulfenylated proteins were detected after exogenous H_2_O_2_ treatment in association with the reduction of APX activities (Figure 7c). Moreover, the abundance of the S‐sulfenylated PbrAPX14 accumulated with the prolonged incubation time of H_2_O_2_, and demonstrated a negative correlation with APX activity (Figure 7d).

Subsequently, we used the *PbrAPX14*‐overexpressing ‘Kolar’ pear and ‘Clapp's Favourite’ fruitlet calli to detect the sulfenylated PbrAPX14 in vivo. As shown in Figure 7e, the sulfenylated PbrAPX14 existed in pear fruit and calli, and its abundance increased after exogenous H_2_O_2_ treatment. Further study revealed that the level of the sulfenylated PbrAPX14 in ‘Kolar’ fruit was upregulated by exogenous ethrel treatment, which promoted endogenous H_2_O_2_ accumulation (Figure S9).

Taken together, our study confirmed that H_2_O_2_‐mediated sulfenylation of the Cys^48^ residue in PbrAPX14, which would weaken its function, took place in vitro and in vivo and partly accounted for the ethylene‐induced H_2_O_2_ accumulation.

### 3.8 Impact of Exogenous H_2_O_2_ Treatment on Ethylene Evolution and Thus Fruit Ripening and Senescence Process

In this study, the positive impact of endogenous H_2_O_2_ on ethylene formation and then the ripening and senescence process was implicated in the phenotype of the *PbrAPX14/PbrIDD2*‐trangenic fruit (Figures 4c,e and 6b,d). Then, we intended to validate such a phenomenon with the aid of exogenous H_2_O_2_ treatment.

As illustrated in Figure S10, exogenous application of H_2_O_2_ considerably upregulated *PbrACS1b*, *PbrACO54*, *PbrERF24* and *PbrERF114* expression levels, ACO and ACS activities, and thus ACC and ethylene production, promoting the ripening and senescence process of ‘Kolar’ pear as demonstrated by the accelerating softening process and colour transition.

## 4 Discussion

Ethylene is a phytohormone involved in the ripening and senescence process of horticultural fruit, causing a series of quality changes (Alba et al. 2005; Jiang et al. 2024). In agreement with the observation in the ripening ‘Anjou’ (Xie et al. 2016) and ‘Housui’ (Jiang et al. 2024) pear, ethylene accumulated during 0°C or 25°C storage of ‘Kolar’ pear with a climacteric peak on 120th day and 10th day, respectively, causing the alternation of colour, chlorophyll a and b, textural and taste qualities (Figures 1a, 2a–i, S1 and S2a,b). Such a phenomenon was associated with the alternation of gene (*PbrACS1b*, *PbrACO54*, *PbrERF24*, *PbrERF114*, etc.) expression profiles, enzyme (ACS and ACO) activity and inter‐metabolite (ACC) content in its biosynthetic pathway (Figure 2a and Table S2). For pear, exogenous 1‐MCP/ethrel treatment or overexpression/mutation of gene involved in ethylene biosynthesis would disturb its metabolism during fruit ripening and senescence process (Hao et al. 2018; Jiang et al. 2024); therefore, these technologies were applied to explore the ethylene‐(in)dependent quality and physio‐biochemical changes (Jiang et al. 2024; Wang, Ma, et al. 2018; Zhang et al. 2019). In combination of the results in Figures 1, 2, S1 and S2a,b, the abovementioned quality attributes in postharvest ‘Kolar’ pear were under the control of ethylene.

Climacteric ethylene is biosynthesized from methionine by the sequential actions of SAMS, ACS and ACO (Alba et al. 2005). In this study, strong correlations were detected among *PbrACO54* mRNA abundance, ACO activity and ethylene evolution in 0°C‐stored ‘Kolar’ pear (control fruit; correlation coefficient > 0.60). On the other hand, the correlations among ethylene content, *PbrACS1b* expression level, and ACS activity were relatively low (control fruit; correlation coefficient < 0.35); however, an extremely strong association was detected between ACS activity and ACC abundance (control fruit; correlation coefficient > 0.80). Besides transcription level, some other factors, such as post‐transcriptional modification (Wang et al. 2015), mRNA translation efficiency (Ying et al. 2025) and post‐translational modification (Waszczak et al. 2014), etc., could impact protein content and thus enzyme activity, which might account for the different expression patterns of *PbrACS1b* mRNA abundance and ACS activity during 0°C storage of ‘Kolar’ pear (control fruit; Figure 2a–ii,iii and Table S2). Therefore, *PbrACO54*, whose function in ethylene biosynthesis has been validated in previous studies of our laboratory (Hao et al. 2018; Wang, Wang, et al. 2018), is selected for further experiment in this study. Previously, Cao et al. (2024) and Wang et al. (2024) have implicated the role of *PbrACS1b* in the ethylene‐mediated ripening process of pear. Consistently, the expression of *PbrACS1b* and then ACS activity in ‘Kolar’ fruit was positively regulated by ethylene as well in this study, with the aid of 1‐MCP‐treated and *PbrACO54*‐overexpressing fruit (Figure 2a–ii,iii,b–ii,iii; Tables S2 and S3). Therefore, we could not eliminate that ACS might participate in ethylene and ROS metabolism in the ripening ‘Kolar’ fruit.

Endogenous ROS, especially H_2_O_2_, proposedly play a crucial role in the ethylene‐dependent ripening and senescence process of horticultural fruit (Masia 2010; Osorio et al. 2011; Dong et al. 2013; Huan et al. 2016; Kumar et al. 2016; Pilati et al. 2014; Wang, Wang, et al. 2018; Gao et al. 2021). Mutation of *Nr*, encoding ethylene receptor 3 (ETR3), resulted in the inhibition of endogenous H_2_O_2_ generation in ‘Ailsa Craig’ tomato (Castagna et al. 2007; Kumar et al. 2016; Osorio et al. 2011). In agreement with the result of Wang, Wang, et al. (2018) using ‘Housui’ fruit as material, the production of endogenous ROS (including H_2_O_2_, ‧OH, and O_2_·^−^) in postharvest ‘Kolar’ pear was positively under the control of ethylene, with the aid of 1‐MCP/ethrel‐treated or *PbrACO54*‐overexpressing fruit (Figures 3a–i,b–i and S2c). Endogenous H_2_O_2_, acting as a secondary messenger in the ethylene signalling pathway, would transcriptionally regulate the expression profiles of the ripening‐related genes or post‐translationally control the function of the ripening‐related proteins, therefore promoting the ripening and senescence of fruit (Guo et al. 2020; Zhou et al. 2025). However, the molecular mechanism responsible for its alternation has not been fully clarified.

In plants, endogenous H_2_O_2_ is under the control of AsA‐GSH cycle (Jiang et al. 2022). Until recently, a bunch of genes (or proteins) have been characterized from various plants to participate in endogenous H_2_O_2_ scavenging, such as a chloroplastic PbDHAR2 from *P. sinkiangensis* (Qin et al. 2015), a chloroplastic SlAPX from *S. lycopersicum* (Duan et al. 2012), a peroxisomal PutAPX from *P. tenuiflora* (Guan et al. 2015), and the cytosolic OsAPX1/2 from *O. sativa* (Rosa et al. 2010). By a conjoint analysis of metabolite, enzyme activities and gene expression profiles in AsA‐GSH cycle followed by experimental validation, a cytosolic PbrAPX14 was explored to function in H_2_O_2_ scavenging in vitro and in vivo and thus the ethylene‐dependent ripening and senescence process of ‘Kolar’ pear (Figures 3, 4, S2c–f, S4, S5a, and S6a; Tables S4–S6). Previously, Kumar et al. (2016) reported that when compared with those in the ripening‐impaired mutant *rin*, lower APX activity in the wild‐type ‘Ailsa Craig’ tomato might be responsible for higher content of H_2_O_2_, which was of great importance for cell wall degradation during fruit ripening.

For horticultural fruit, the expression of the structural gene is under the control of TFs (Jia et al. 2024). PbMYB5, an upstream regulator of *PbDHAR2*, could bind to the MYB recognition site (core motif: CAACTG) to initiate its transcription, mitigating endogenous H_2_O_2_ accumulation and thus chilling sensitivity of *P. betulaefolia* (Xing et al. 2019). Similarly, a C_2_H_2_‐type zinc finger transcription factor from *Populus euphratica*, PeSTZ1, enhanced freezing tolerance of 84 K poplar through modulation of endogenous H_2_O_2_‐scavenging capacity by directly controlling *PeAPX2* expression (He et al. 2019). In this study, the nucleus‐located PbrIDD2, whose expression was negatively regulated by ethylene, was characterized and validated to directly interact with the *PbrAPX14* promoter to activate its expression, inhibit endogenous H_2_O_2_ accumulation and thus suppress the ripening and senescence process of fruit (Figures 5, 6, S2e,f, S5b, S6b, and S7; Tables S7 and S8). In a previous study, Su et al. (2019) reported that *PbIDD2*, which was mainly expressed in fruit, flower and bud of *P. bretschneideri*, might participate in the gibberellin flowering pathway during physiological flower bud differentiation and regulate the plant flowering process in response to sugar and photoperiod signals. Additionally, MaC2H2‐IDD in ‘Fenjiao’ banana promoted the fruit ripening and softening process via binding to the promoters of genes involved in cell wall and starch degradation and then modulating their activities (Song et al. 2024). Thus, our study extended the function of IDD family gene during fruit ripening.

Besides the transcriptional regulation, a bunch of the post‐translational modifications of proteins, creating a complex landscape of protein diversity and function, have been discovered in the plant kingdom (Waszczak et al. 2014). For example, H_2_O_2_ could oxidize the proteinaceous cysteinyl thiol to sulfenic acid, known as S‐sulfenylation (Cys‐SOH), thereby affecting protein stability, conformation, activity, interaction and subcellular localization (Huang et al. 2019). This post‐translational modification participates in plant development (Sun et al. 2025) as well as response to abiotic stress (Waszczak et al. 2014; Huang et al. 2019). A total of 1537 S‐sulfenylated sites had been mapped from 1394 proteins in *Arabidopsis* (Huang et al. 2019). In combination of *in silico* analysis and experimental validation, the Cys^48^ residue in PbrAPX14, which was conserved in plant cytosolic/chloroplastic APXs for AsA binding (Kitajima 2008; Kitajima et al. 2008; Chin et al. 2019), could be S‐sulfenylated by H_2_O_2_ in vitro and in vivo, therefore mitigating its function to facilitate the ethylene‐induced endogenous H_2_O_2_ accumulation (Figures 7 and S9). A similar phenomenon was also observed after exogenous H_2_O_2_ treatment of a cytosolic APX from *Pisum sativum* (PsAPX) (Hiner et al. 2000), a chloroplastic APX from *Spinacia oleracea* (SoAPX) (Miyake and Asada 1996), or a chloroplastic NtAPX from *N. tabacum* (Kitajima et al. 2008). In combination of the outcomes from previous studies (Miyake and Asada 1996; Kitajima et al. 2008), the H_2_O_2_‐induced inactivation of PbrAPX14 might be due to its S‐sulfenylation of the Cys^48^ residue in the two‐electron‐oxidized intermediate of PbrAPX14 (named as PbrAPX14 (Fe^VI^=O)R·) in association with the degradation of heme moiety (Figure S11). PbrAPX14 (Fe^VI^=O)R· is derived from the oxidation of PbrAPX14 (named as PbrAPX14 (Fe^III^)R) by H_2_O_2_, in which the heme moiety is oxidized to the oxyferryl (Fe^IV^=O) species and an organic group (R), either the porphyrin or the side chain of an amino acid, is oxidized to a free radical (R·) (Figure S11) (Miyake and Asada 1996).

In our study, the positive role of endogenous H_2_O_2_ in ethylene formation and then the ripening and senescence process of ‘Kolar’ pear was implicated after overexpression of *PbrAPX14* and *PbrIDD2* genes or exogenous H_2_O_2_ treatment (Figures 4c,e, 6b,d and S10), forming a positive feedback. Consistent with this, 10 mM H_2_O_2_ treatment of ‘Verty F1’ tomato facilitated ethylene production, colour development, water‐soluble pectin accumulation and firmness decrement (Torun and Uluisik 2022). A similar phenomenon was observed in ‘Bartlett’ pear (Brennan and Frenkel 1977), ‘Ailsa Craig’ tomato (Zhou et al. 2025), ‘Fuyan’ longan (Lin et al. 2014; Lin et al. 2020) and ‘Hongshi’ kiwifruit (Yan et al. 2023) after exogenous treatment of H_2_O_2_ or its substrates, where quality change (colour transition, softening process, etc.) and/or ethylene evolution was promoted when compared with the controls. Furthermore, the elevation of ethylene evolution in ‘Kolar’ fruit might be due to the H_2_O_2_‐mediated alternation of gene expression profile in its biosynthetic pathway (Figure S10c). When compared with that in control fruit, the regulated *SlACS4* transcripts at early ripening stage might account for higher ethylene level in the H_2_O_2_‐treated ‘Verty F1’ tomato (Torun and Uluisik 2022). Consistently, the expression levels of *LcACS1*, *LcACO2* and *LcEIL3* genes in the fruitlet abscission zone of litchi were suppressed after silence of *respiratory burst oxidase homologue D* (*LcRbohD*), which was responsible for endogenous O_2_^−^· and H_2_O_2_ generation (Hong et al. 2020; Ma et al. 2023).

**FIGURE 8:**
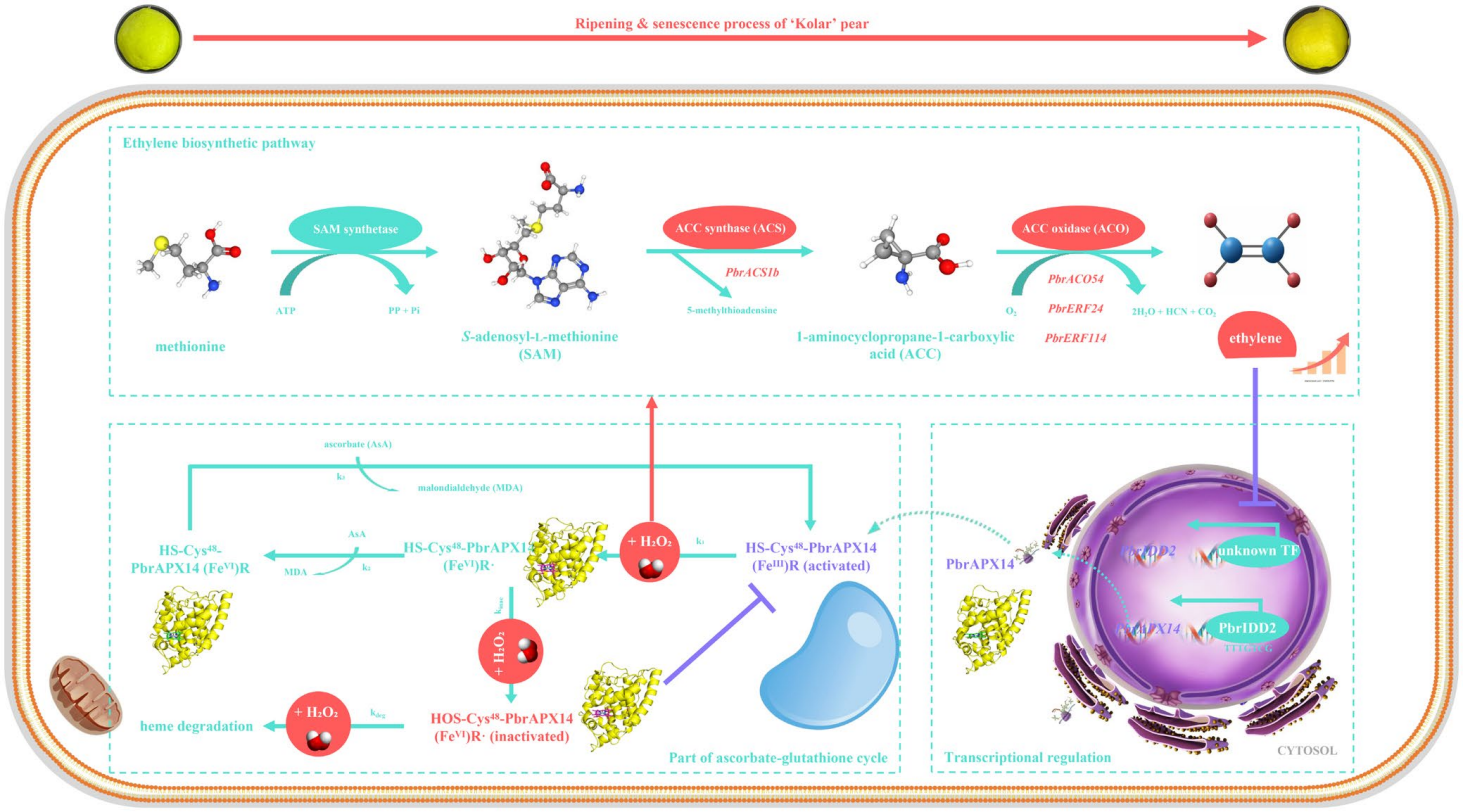
Schematic model on the role of PbrIDD2‐*PbrAPX14* module in the ethylene‐mediated ripening and senescence process of pear. During ‘Kolar’ pear storage, ethylene accumulated with a climacteric peak, which was associated with the (incipient) increment of gene (*PbrACS1b*, *PbrACO54*, *PbrERF24*, *PbrERF114*, etc.) transcripts, enzyme (ACO and ACS) activities and ACC content in its biosynthetic pathway. However, the expression abundances of *PbrIDD2* and its downstream structural gene *PbrAPX14* were downregulated by ethylene. PbrIDD2, located in the nucleus, could interact with the *cis*‐acting element (core motif: TTTGTCG) in *PbrAPX14* promoter and then activate its expression; after translation in ribosome, PbrAPX14 was then transported into cytosol, where it reduced H_2_O_2_ into H_2_O. The downregulated mRNA abundances of *PbrIDD2* and *PbrAPX14* promoted endogenous H_2_O_2_ accumulation and then fruit ripening and senescence process. Additionally, H_2_O_2_ would post‐translationally S‐sulfenylate Cys^48^ residue in PbrAPX14 (maybe the two‐electron‐oxidized intermediate of PbrAPX14, named as PbrAPX14 (Fe^VI^=O)R·) in association with the degradation of heme moiety, which would mitigate APX activity to facilitate endogenous H_2_O_2_ accumulation. Moreover, H_2_O_2_ played a positive role in ethylene metabolism in postharvest fruit, forming a positive feedback.

Taken together, this study uncovered the role of H_2_O_2_ in the ethylene‐mediated ripening and senescence process of pear; thus, exogenous H_2_O_2_ treatment could be applied in practical postharvest treatment and management to promote fruit maturity. Moreover, *PbrIDD2* and *PbrAPX14*, which were responsible for the dynamic change of H_2_O_2_ level during pear ripening and senescence, could be acted as gene source for future breeding program to generate fruit with longer marketing time. A schematic model on the role of PbrIDD2‐*PbrAPX14* module in the ethylene‐mediated ripening and senescence process of pear was proposed as illustrated in Figure 8. During ‘Kolar’ pear storage, ethylene accumulated with a climacteric peak, which was associated with the (incipient) increment of gene (*PbrACS1b*, *PbrACO54*, *PbrERF24*, *PbrERF114*, etc.) transcripts, enzyme (ACO and ACS) activities and ACC content in its biosynthetic pathway. However, the expression abundances of *PbrIDD2* and its downstream structural gene *PbrAPX14* were downregulated by ethylene. PbrIDD2, located in the nucleus, could interact with the *cis*‐acting element (core motif: TTTGTCG) in *PbrAPX14* promoter and then activate its expression; after translation in ribosome, PbrAPX14 was then transported into cytosol, where it reduced H_2_O_2_ into H_2_O. The downregulated mRNA abundances of *PbrIDD2* and *PbrAPX14* promoted endogenous H_2_O_2_ accumulation and then fruit ripening and senescence process. Additionally, H_2_O_2_ would post‐translationally S‐sulfenylate Cys^48^ residue in PbrAPX14 (maybe the two‐electron‐oxidized intermediate of PbrAPX14, named as PbrAPX14 (Fe^VI^=O)R**·**) in association with the degradation of heme moiety, which would mitigate APX activity to facilitate endogenous H_2_O_2_ accumulation. Moreover, H_2_O_2_ played a positive role in ethylene metabolism in postharvest fruit, forming a positive feedback.

## 5 Conclusion

During ‘Kolar’ pear storage, ethylene, which accumulated with a climacteric peak, suppressed the transcription of *PbrIDD2* and its downstream structural gene *PbrAPX14*. The abovementioned phenomenon promoted H_2_O_2_ accumulation, and thus fruit ripening and senescence process. In addition, H_2_O_2_ could post‐translationally S‐sulfenylated Cys^48^ residue in PbrAPX14 in vitro and in vivo, which mitigated its function and therefore facilitated the ethylene‐induced accumulation of endogenous H_2_O_2_. Moreover, H_2_O_2_ would promote ethylene evolution in postharvest fruit, forming a positive feedback.

Taken together, both transcriptional regulation of *PbrAPX14* mRNA abundance by PbrIDD2 and post‐translational modification of PbrAPX14 protein function by H_2_O_2_, which were (in)directly regulated by ethylene, might account for the dynamic equilibrium of H_2_O_2_ level in postharvest pear, finally influencing fruit ripening and senescence process.

## Funding

This work was financially supported by the Major Scientific and Technological Project of Xinjiang (Grant No. 2024A02006), the Natural Science Foundation of Guangxi (Grant No. 2022JJA130045), the Municipal Science and Technology Project of Alar (Xinjiang) in 2022 (Grant No. 2022XX5), the National Natural Science Foundation of China (Grant No. 32302615, 31872070, 31830081 and 31701868), the China Postdoctoral Science Foundation (Grant No. 2025M773739), the Jiangsu Agricultural Science and Technology Innovation Fund (Grant No. CX(24)1024), the Zhongshan Biological Breeding Laboratory (Grant No. ZSBBL‐KY2024‐03), the Priority Academic Program Development of Jiangsu Higher Education Institutions, the Earmarked Fund for Agriculture Research System of China (Grant No. CARS‐28).

## Conflicts of Interest

The authors declare no conflicts of interest.

## Supporting information


**Figure S1:** Impact of exogenous 1‐MCP treatment on chlorophyll a and b contents in ‘Kolar’ pear. ‘Kolar’ pears were treated with H_2_O (control) and 1‐MCP before storage at 0°C for 180 days; fruits were sampled every 60 days. Data represent the mean value of three biological replicates, and significant difference (*p < 0.05; **p < 0.01; ***p < 0.001) between samples at the same sampling time is determined via one‐way ANOVA.
**Figure S2:** Impact of exogenous 1‐MCP and ethrel treatments on quality and physiol‐biochemical attributes and gene expression profiles during 20°C storage of ‘Kolar’ pear. (a) Firmness. (b) Ethylene evolution. (c) H_2_O_2_ content. (d) APX activity. (e) *PbrAPX14* and *PbrIDD2* expression abundances. (f) Correlation between attributes. ‘Kolar’ pears were treated with H_2_O (control), ethrel and 1‐MCP before 20°C storage for 20 days; fruits were sampled every 10 days. The expression level of each gene at 0th day is set as 1.0 for qRT‐PCR assay. Data represent the mean value of three biological replicates, and significant difference (*p < 0.05; **p < 0.01; ***p < 0.001) between samples at the same sampling time is determined via one‐way ANOVA. Spearman correlation between different attributes is visualized in the heatmap, where red colour demonstrates positive correlation, while purple colour indicates negative association.
**Figure S3:** qRT‐PCR validation of transcriptome results on gene expression profiles. (a) Exogenous 1‐MCP treatment. (a‐i) Genes involved in ethylene biosynthesis. (a‐ii) Genes in AsA‐GSH cycle. ‘Kolar’ pears were treated with H_2_O (control) and 1‐MCP before storage at 0°C for 180 days; fruits were sampled every 60 days. The expression level of each gene at 0th day is set as 1.0 for qRT‐PCR assay. (b) Transient overexpression of *PbrACO54* gene. (b‐i) Genes involved in ethylene biosynthesis. (b‐ii) Genes in AsA‐GSH cycle. ‘Kolar’ pear transformed with the empty pCAMBIA1301 vector was used as the control for the OE fruit. After infiltration, fruits were preserved at 20°C for 5 days before sampling. The expression level of each gene in the control fruit is set as 1.0 for qRT‐PCR assay. Data represent the mean value of three biological replicates, and significant difference (*p < 0.05; **p < 0.01; ***p < 0.001) between samples at the same sampling time is determined via one‐way ANOVA.
**Figure S4:** Functional validation of PbrAPX14 in vitro. (a) Alignment of cytosolic APXs in plants. Information on the cytosolic APXs in plants was summarized in Table S6, and the AsA‐binding residues were highlighted in the boxes based on the report of Chin et al. (2019). (b) Michaelis‐Menten curve for AsA conversion by PbrAPX14 in vitro.
**Figure S5:** Signal peptide and transmembrane helice in protein. (a) PbrAPX14. (a‐i) Signal peptide. (a‐ii) Transmembrane helice. (b) PbrIDD2. (b‐i) Signal peptide. (b‐ii) Transmembrane helice.
**Figure S6:** Identification of the transgenic lines by western blotting. (a) *PbrAPX14*‐overexpressing pear calli (a‐i) and tomato fruit (a‐ii). (b) *PbrIDD2*‐overexpressing pear calli (b‐i) and tomato fruit (b‐ii). Calli transformed with the empty pCAMBIA1301 vector was used as the control for the OE calli (after transformation, calli was grown at 20°C); on the other hand, the wide‐type tomato was used as the control for the OE fruit (all plants were grown in the greenhouse (light for 18 h at 25°C and dark for 6 h at 18°C, 60% RH) before sampling tomato fruit at 44 DAFB). An anti‐GFP antibody was used to detect the abundances of PbrAPX14‐GFP and PbrIDD2‐GFP, while an anti‐actin antibody was used to detect the amount of β‐actin.
**Figure S7:** Identification of the possible upstream activators of *PbrAPX14* gene. (a) Information on the differentially expressed TFs after exogenous 1‐MCP treatment of ‘Kolar’ pear. (a‐i) Venn diagram representing the number of the differentially expressed TFs per stage and their intersection with others. The differentially expressed TFs were identified based on the following criteria: fold change ≥ 2.0 and *p*adj < 0.05. (a‐ii) Distribution of 456 differentially expressed TFs, whose mRNA abundances were consistently higher (or lower) in the 1‐MCP‐treated fruit than those in the control fruit throughout storage. ‘Kolar’ pears were treated with H_2_O (control) and 1‐MCP before storage at 0°C for 180 days; fruits were sampled every 60 days. (b) Expression profiles of 456 differentially expressed TFs and their correlations with *PbrAPX14* gene after exogenous 1‐MCP treatment of ‘Kolar’ pear. Data, adapted from transcriptome assay, represent the mean value of three biological replicates. Colour scale represents normalized log_2_‐transformed (mean FPKM +1), where cyan indicates a high level, purple presents a low level and white demonstrates a medium level. Spearman correlation between different attributes is visualized in the heatmap, where cyan (or light cyan) line demonstrates extreme strong (or strong) positive correlation, while purple (or light purple) line indicates extreme strong (or strong) negative association. (c) Expression profiles of 40 differentially expressed TFs after transient overexpression of *PbrACO54* gene in ‘Kolar’ pear. ‘Kolar’ pear transformed with the empty pCAMBIA1301 vector was used as the control for the OE fruit. After infiltration, fruits were preserved at 20°C for 5 days before sampling. Forty differentially expressed TFs were identified based on the following criteria: their expression levels were upregulated by 1‐MCP treatment throughout 0°C storage (fold change ≥ 2.0 and *p*adj < 0.05 for at least one stage), and demonstrated extreme strong positive correlations with *PbrAPX14* transcripts (correlation coefficient ≥ 0.8; Figure S7b and Table S7). Data, adapted from transcriptome assay, represent the value of three biological replicates. Colour scale represents normalized log_2_‐transformed (FPKM +1), where red indicates a high level, cyan presents a low level and white demonstrates a medium level. (d) Detailed information on the PbrIDD2‐binding element in *PbrAPX14* promoter. The PbrIDD2‐binding element (core motif: TTTGTCG) in *PbrAPX14* promoter was predicted with the aid of the PlantRegMap database (Tian et al. 2020).
**Figure S8:** Alternation of APX activity after mutation of cysteine residue at position 48 (Cys^48^) in PbrAPX14 with serine residue (Ser^48^). Structures of the wild‐type PbrAPX14 and its mutant (PbrAPX14^Cys48Ser^) were predicted by Modeller 10.6 software (Webb and Sali 2016). The activity of the wild‐type PbrAPX14 was set as 1.0.
**Figure S9:** Impact of exogenous ethrel treatment on the abundance of the S‐sulfenylated PbrAPX14 in pear fruit. (a) Ethylene evolution and H_2_O_2_ level. (b) Abundance of the sulfenylated PbrAPX14. After transformation with *PbrAPX14*, ‘Kolar’ pears were divided into two groups for H_2_O (control) and 0.5 mL L^−1^ ethrel treatments. Fruits were sampled after 20°C storage for 5 days. Relative abundance of the S‐sulfenylated PbrAPX14 in vivo was calculated as the ratio of the S‐sulfenylated PbrAPX14‐Flag and loading control (PbrAPX14‐Flag) in the sample. Data represent the mean value of three biological replicates, and significant difference (*p < 0.05; **p < 0.01; ***p < 0.001) between samples is determined via one‐way ANOVA.
**Figure S10:** Impact of exogenous H_2_O_2_ treatment on ethylene metabolism and physiological and quality attributes during 20°C storage of ‘Kolar’ pear. (a) Visual quality. (b) Endogenous H_2_O_2_. (c) Ethylene metabolism. (d) Quality attributes. ‘Kolar’ pears after harvest were treated with H_2_O (control) and H_2_O_2_ before 20°C storage for 20 days; fruits were sampled every 10 days. Data represent the mean value of three biological replicates, and significant difference (*p < 0.05; **p < 0.01; ***p < 0.001) between samples is determined via one‐way ANOVA.
**Figure S11:** Schematic model on the catalytic cycle of PbrAPX14 and inactivation of its function by H_2_O_2_. The model was drawn in combination of the outcomes from previous studies (Miyake and Asada 1996; Kitajima et al. 2008). Structure of the wild‐type PbrAPX14 was predicted by Modeller 10.6 software (Webb and Sali 2016).

**Table S1:** Primers used in this study.
**Table S2:** Impact of exogenous 1‐MCP treatment on gene expression profiles in ethylene biosynthetic pathway during 0°C storage of ‘Kolar’ pear. ‘Kolar’ pears were treated with H_2_O (control) and 1‐MCP before storage at 0°C for 180 days; fruits were sampled every 60 days. Data, adapted from transcriptome assay, represented the mean values of three biological replicates.
**Table S3:** Alternation of gene expression profiles in ethylene biosynthetic pathway after transient overexpression of *PbrACO54* gene in ‘Kolar’ pear. ‘Kolar’ pear transformed with the empty pCAMBIA1301 vector was used as the control for the OE fruit. After infiltration, fruits were preserved at 20°C for 5 days before sampling. Data, adapted from transcriptome assay, represented the value of three biological replicates.
**Table S4:** Impact of exogenous 1‐MCP treatment on gene expression profiles in AsA‐GSH cycle during 0°C storage of ‘Kolar’ pear. ‘Kolar’ pears were treated with H_2_O (control) and 1‐MCP before storage at 0°C for 180 days; fruits were sampled every 60 days. Data, adapted from transcriptome assay, represented the mean values of three biological replicates.|
**Table S5:** Alternation of gene expression profiles in AsA‐GSH cycle after transient overexpression of *PbrACO54* gene in ‘Kolar’ pear. ‘Kolar’ pear transformed with the empty pCAMBIA1301 vector was used as the control for the OE fruit. After infiltration, fruits were preserved at 20°C for 5 days before sampling. Data, adapted from transcriptome assay, represented the value of three biological replicates.
**Table S6:** Information on APXs in plants.
**Table S7:** Impact of exogenous 1‐MCP treatment on the differentially expressed TFs during 0°C storage of ‘Kolar’ pear. ‘Kolar’ pears were treated with H_2_O (control) and 1‐MCP before storage at 0°C for 180 days; fruits were sampled every 60 days. A total of 456 differentially expressed TFs, whose mRNA abundances were consistently higher (or lower) in the 1‐MCP‐treated fruit than those in the control fruit throughout storage, were identified by DESeq software based on the following criteria: fold change ≥ 2.0 and *p*adj < 0.05 for at least one stage. Data, adapted from transcriptome assay, represented the mean values of three biological replicates.
**Table S8:** Alternation in gene expression profiles of 40 differentially expressed TFs after transient overexpression of *PbrACO54* gene in ‘Kolar’ pear. ‘Korla’ pear transformed with the empty pCAMBIA1301 vector was used as the control for the OE fruit. After infiltration, fruits were preserved at 20°C for 5 days before sampling. 40 TFs were identified based on the following criteria: their expression levels were upregulated by 1‐MCP treatment throughout 0°C storage (fold change ≥ 2.0 and *p*adj < 0.05 for at least one stage), and demonstrated extreme strong positive correlations with *PbrAPX14* transcripts (correlation coefficient ≥ 0.8; Figure S7b and Table S7). Data, adapted from transcriptome assay, represented three biological replicates.

## Data Availability

Transcriptome assay was conducted with the aid of Novogene Technology Co. (Beijing, China) on the Illumina NovaSeq 6000 platform. The raw sequence data, which was reported in this paper, has been deposited in the NCBI Sequence Read Archive (SRA) database (BioProject ID: PRJNA1240921). All data generated and analysed during this study were included in this published article and its Supporting Information files (Tables S2–S5, S7 and S8). Moreover, all other data are available from the corresponding author upon reasonable request.
