# Supplementary material for: PbrIDD2‐ PbrAPX14 Module Functions in the Ethylene‐Mediated Ripening and Senescence Process of Pear Fruit
Source: Plant Biotechnol J. 2026 Apr 27;24(8):4878–96. doi: 10.1111/pbi.70664 (PMC13387890; doi:10.1111/pbi.70664)
Supplement: Supplementary file 1 — Figure S1: Impact of exogenous 1‐MCP treatment on chlorophyll a and b contents in ‘Kolar’ pear. ‘Kolar’ pears were treated with H2O (control) and 1‐MCP before storage at 0°C for 180 days; fruits were sampled every 60 days. Data represent the mean value of three biological replicates, and significant difference (*p < 0.05; **p < 0.01; ***p < 0.001) between samples at the same sampling time is determined via one‐way ANOVA. Figure S2: Impact of exogenous 1‐MCP and ethrel treatments on quality and physiol‐biochemical attributes and gene expression profiles during 20°C storage of ‘Kolar’ pear. (a) Firmness. (b) Ethylene evolution. (c) H2O2 content. (d) APX activity. (e) PbrAPX14 and PbrIDD2 expression abundances. (f) Correlation between attributes. ‘Kolar’ pears were treated with H2O (control), ethrel and 1‐MCP before 20°C storage for 20 days; fruits were sampled every 10 days. The expression level of each gene at 0th day is set as 1.0 for qRT‐PCR assay. Data represent the mean value of three biological replicates, and significant difference (*p < 0.05; **p < 0.01; ***p < 0.001) between samples at the same sampling time is determined via one‐way ANOVA. Spearman correlation between different attributes is visualized in the heatmap, where red colour demonstrates positive correlation, while purple colour indicates negative association. Figure S3: qRT‐PCR validation of transcriptome results on gene expression profiles. (a) Exogenous 1‐MCP treatment. (a‐i) Genes involved in ethylene biosynthesis. (a‐ii) Genes in AsA‐GSH cycle. ‘Kolar’ pears were treated with H2O (control) and 1‐MCP before storage at 0°C for 180 days; fruits were sampled every 60 days. The expression level of each gene at 0th day is set as 1.0 for qRT‐PCR assay. (b) Transient overexpression of PbrACO54 gene. (b‐i) Genes involved in ethylene biosynthesis. (b‐ii) Genes in AsA‐GSH cycle. ‘Kolar’ pear transformed with the empty pCAMBIA1301 vector was used as the control for the OE fruit. After infiltration, fruits [file PBI-24-4878-s001.pdf]

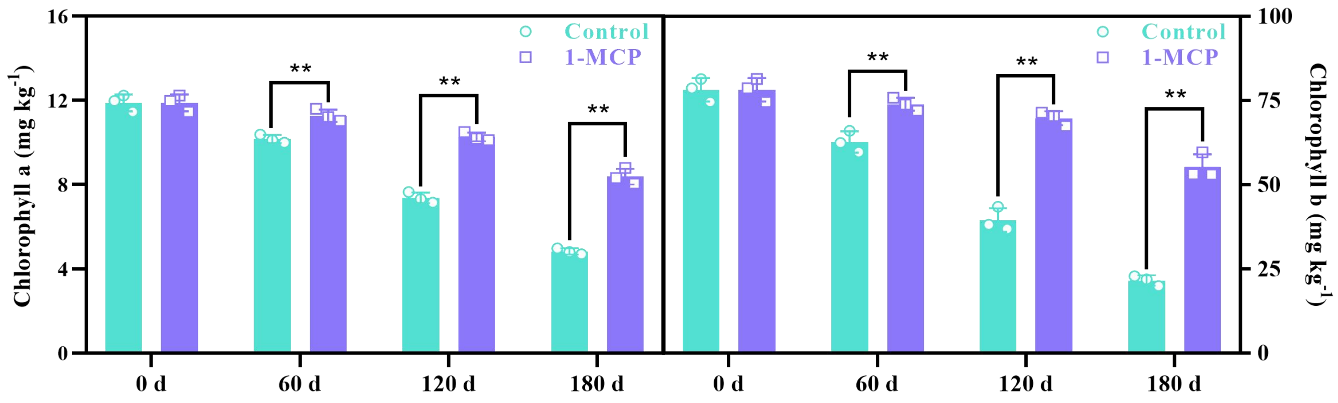

Chlorophyll a (mg kg-1)
16
12
8
4
0
Control
1-MCP
**
**
**
0 d
60 d
120 d
180 d
Chlorophyll b (mg kg-1)
100
75
50
25
0
Control
1-MCP
**
**
**
0 d
60 d
120 d
180 d

FIGURE S1

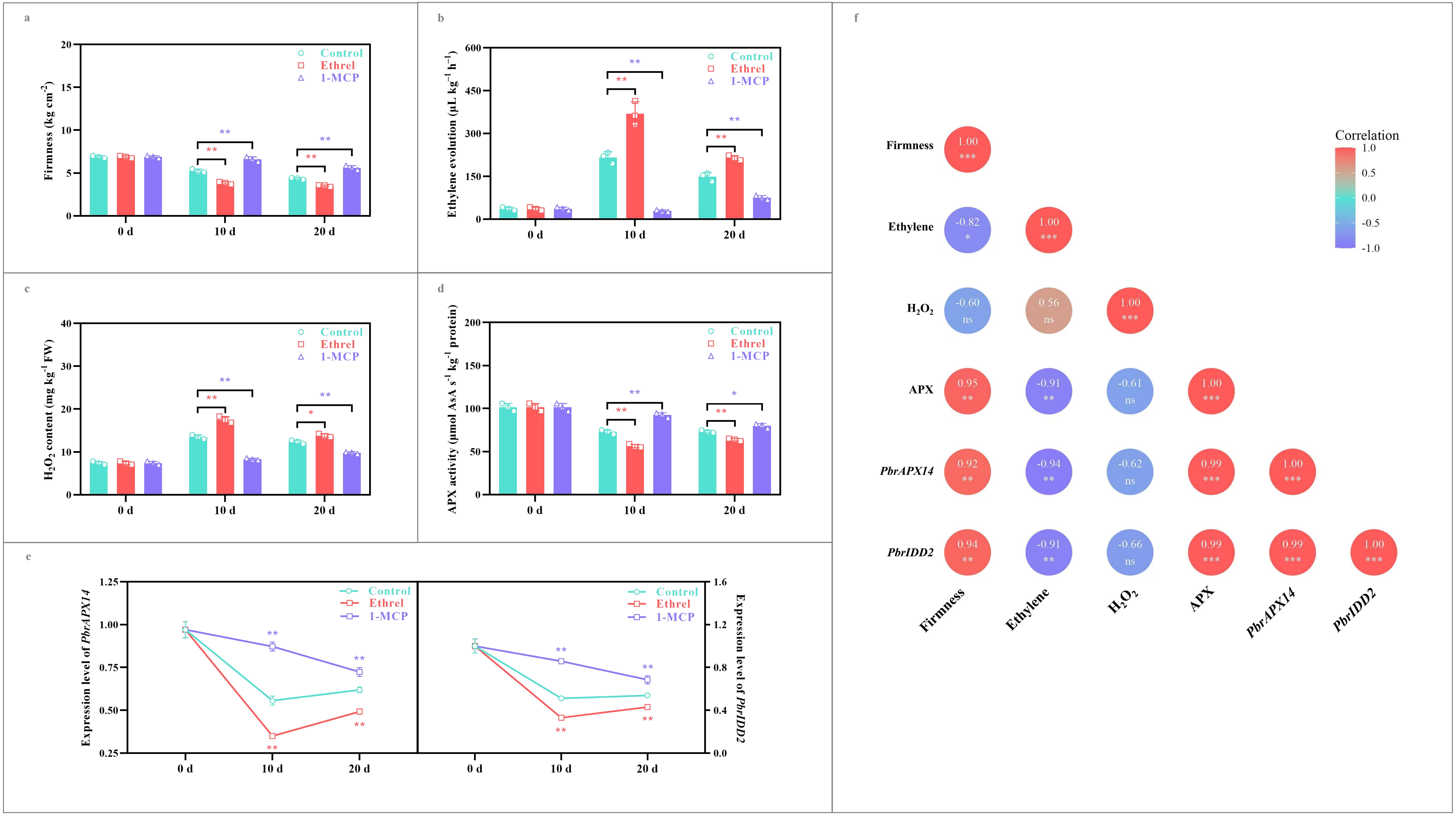
a
Firmness (kg cm^{-2})
Control
Ethrel
1-MCP
0 d
10 d
20 d
b
Ethylene evolution (μL kg^{-1} h^{-1})
c
H_2O_2 content (mg kg^{-1} FW)
d
APX activity (μmol AsA s^{-1} kg^{-1} protein)
e
Expression level of PbrAPX14
Expression level of PbrIDD2
f
Correlation
Firmness
Ethylene
H_2O_2
APX
PbrAPX14
PbrIDD2

FIGURE S2

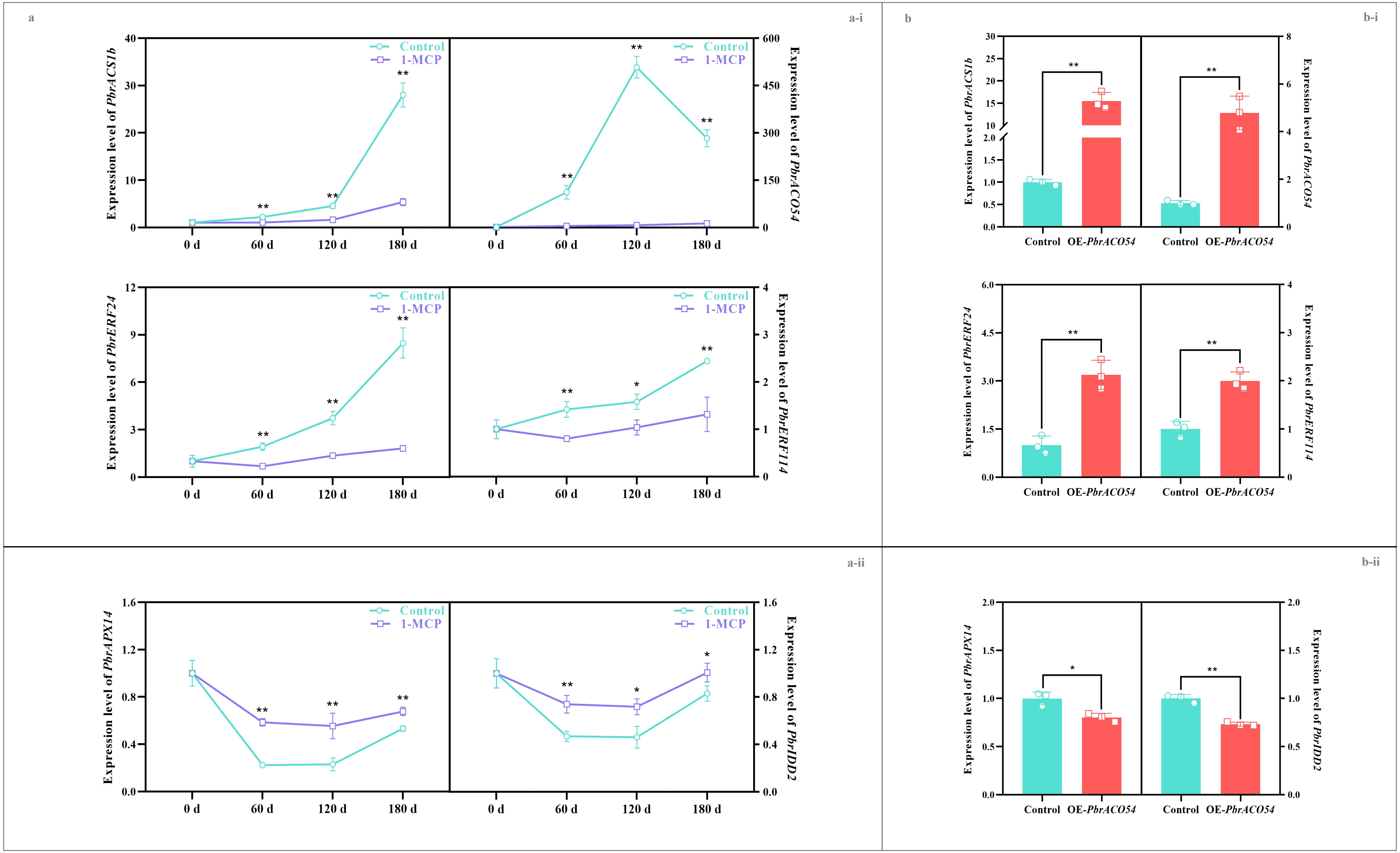
a
a-i
b
b-i
a-ii
b-ii
Control
1-MCP
Expression level of PbrACS1b
Expression level of PbrACO54
Expression level of PbrERF24
Expression level of PbrERF114
Expression level of PbrAPX14
Expression level of PbrIDD2
0 d
60 d
120 d
180 d
Control
OE-PbrACO54

FIGURE S3

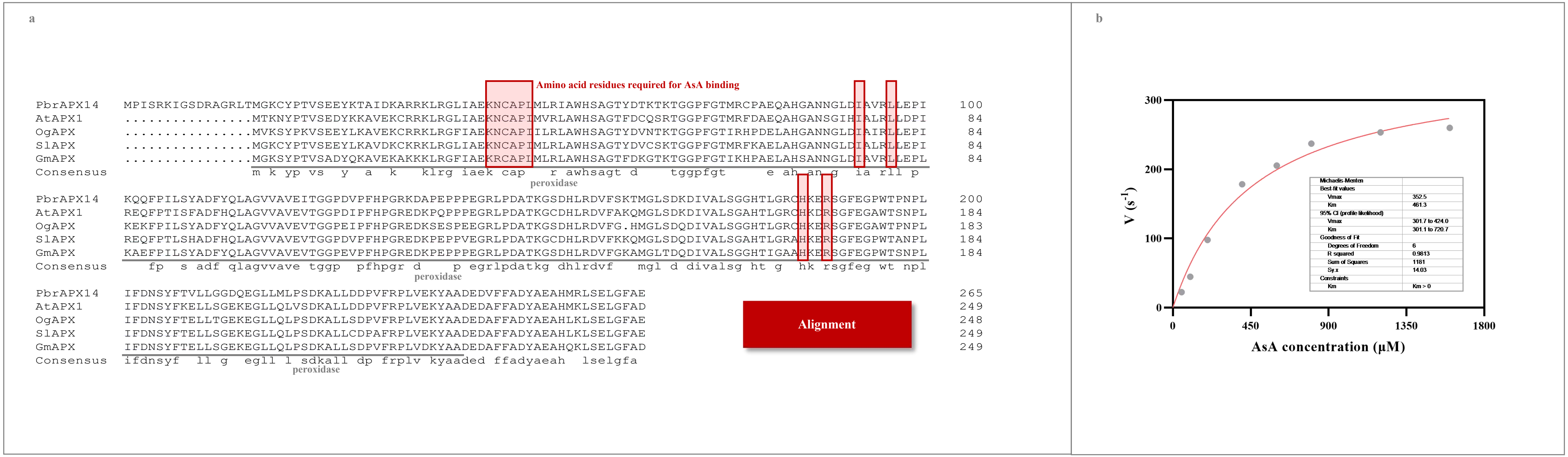
a
Amino acid residues required for AsA binding
PbrAPX14 MPISRKIGSDRAGRLTMGKCYPTVSEEYKTAIDKARRKLRGLIAEKNCAPIMLRIAWHSAGTYDTKTKTGGPFGTMRCPAEQAHGANNGLDIAVRLLEPI 100
AtAPX1 ................MTKNYPTVSEDYKKAVEKCRRKLRGLIAEKNCAPIMVRLAWHSAGTFDCQSRTGGPFGTMRFDAEQAHGANSGIHIALRLLDPI 84
OgAPX ................MVKSYPKVSEEYLKAVEKCRRKLRGFIAEKNCAPIILRLAWHSAGTYDVNTKTGGPFGTIRHPDELAHGANNGLDIAIRLLEPI 84
SlAPX ................MGKCYPTVSEEYLKAVDKCKRKLRGLIAEKNCAPIMLRLAWHSAGTYDVCSKTGGPFGTMRFKAELAHGANNGLDIALRLLEPI 84
GmAPX ................MGKSYPTVSADYQKAVEKAKKKLRGFIAEKRCAPIMLRLAWHSAGTFDKGTKTGGPFGTIKHPAELAHSANNGLDIAVRLLEPL 84
Consensus m k yp vs y a k klrg iaek cap r awhsagt d tggpfgt e ah an g ia rll p
peroxidase
PbrAPX14 KQQFPILSYADFYQLAGVVAVEITGGPDVPFHPGRKDAPEPPPEGRLPDATKGSDHLRDVFSKTMGLSDKDIVALSGGHTLGRCHKERSGFEGPWTPNPL 200
AtAPX1 REQFPTISFADFHQLAGVVAVEVTGGPDIPFHPGREDKPQPPPEGRLPDATKGCDHLRDVFAKQMGLSDKDIVALSGAHTLGRCHKDRSGFEGAWTSNPL 184
OgAPX KEKFPILSYADFYQLAGVVAVEVTGGPEIPFHPGREDKSESPEEGRLPDATKGSDHLRDVFG.HMGLSDQDIVALSGGHTLGRCHKERSGFEGAWTPNPL 183
SlAPX REQFPTLSHADFHQLAGVVAVEVTGGPDVPFHPGREDKPEPPVEGRLPDATKGCDHLRDVFKKQMGLSDQDIVALSGAHTLGRAHKERSGFEGPWTANPL 184
GmAPX KAEFPILSYADFYQLAGVVAVEVTGGPEVPFHPGREDKPEPPPEGRLPDATKGSDHLRDVFGKAMGLTDQDIVALSGGHTIGAAHKERSGFEGPWTSNPL 184
Consensus fp s adf qlagvvave tggp pfhpgr d p egrlpdatkg dhlrdvf mgl d divalsg ht g hk rsgfeg wt npl
peroxidase
PbrAPX14 IFDNSYFTVLLGGDQEGLLMLPSDKALLDDPVFRPLVEKYAADEDVFFADYAEAHMRLSELGFAE 265
AtAPX1 IFDNSYFKELLSGEKEGLLQLVSDKALLDDPVFRPLVEKYAADEDAFFADYAEAHMKLSELGFAD 249
OgAPX IFDNSYFTELLTGEKEGLLQLPSDKALLSDPVFRPLVEKYAADEDAFFADYAEAHLKLSELGFAE 248
SlAPX IFDNSYFTELLSGEKEGLLQLPSDKALLCDPAFRPLVEKYAADEDAFFADYAEAHLKLSELGFAE 249
GmAPX IFDNSYFTELLSGEKEGLLQLPSDKALLSDPVFRPLVDKYAADEDAFFADYAEAHQKLSELGFAD 249
Consensus ifdnsyf ll g egll l sdkall dp frplv kyaaded ffadyaeah lselgfa
peroxidase
Alignment
b
300
200
100
0
V (s-1)
0
450
900
1350
1800
AsA concentration (μM)
Michaelis-Menten
Best-fit values
Vmax 352.5
Km 461.3
95% CI (profile likelihood)
Vmax 301.7 to 424.0
Km 301.1 to 720.7
Goodness of Fit
Degrees of Freedom 6
R squared 0.9813
Sum of Squares 1181
Sy.x 14.03
Constraints
Km Km > 0

FIGURE S4

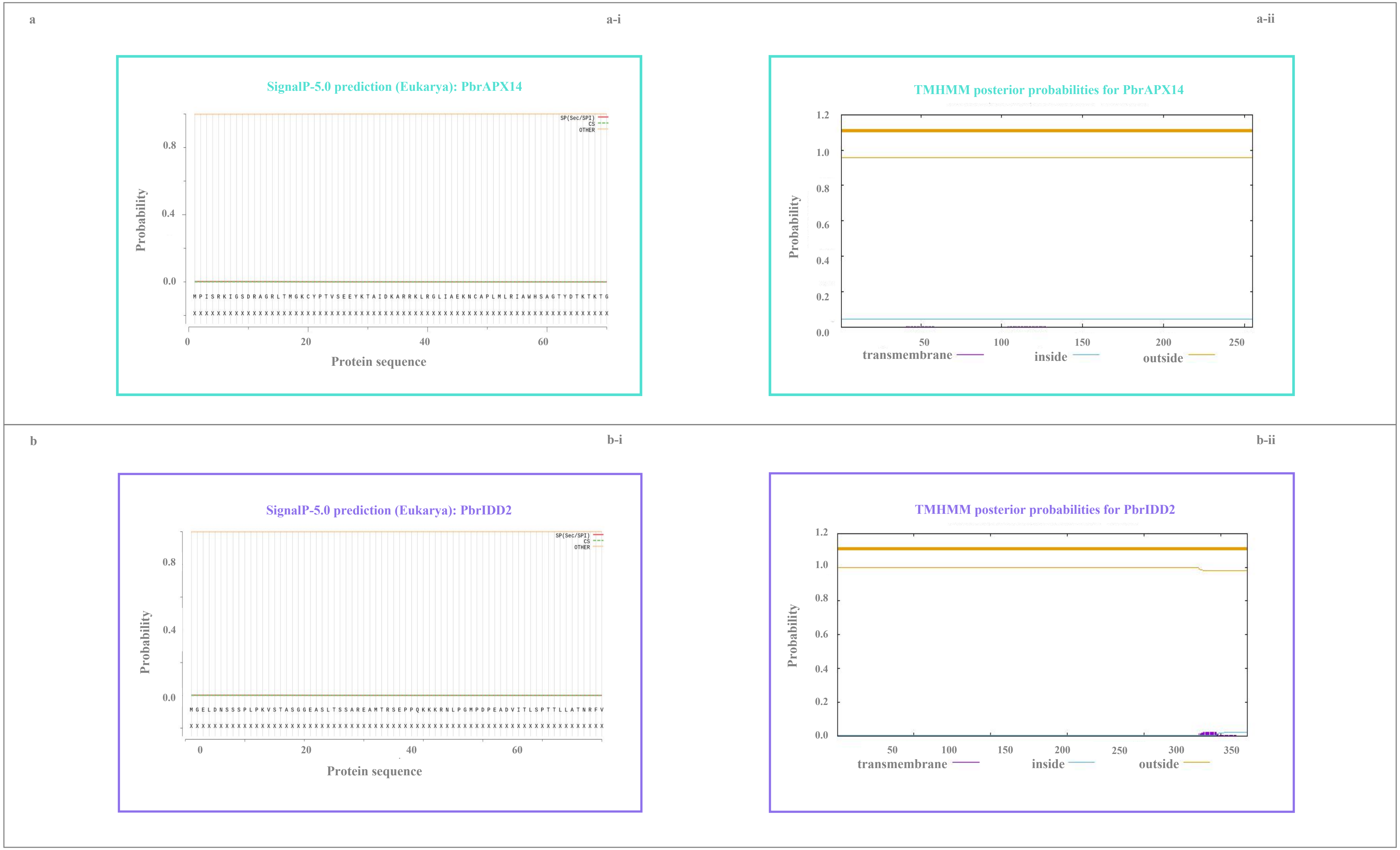
a
a-i
a-ii
SignalP-5.0 prediction (Eukarya): PbrAPX14
SP(Sec/SPI)
CS
OTHER
0.8
0.4
0.0
Probability
M P I S R K I G S D R A G R L T M G K C Y P T V S E E Y K T A I D K A R R K L R G L I A E K N C A P L M L R I A W H S A G T Y D T K T K T G
0
20
40
60
Protein sequence
TMHMM posterior probabilities for PbrAPX14
1.2
1.0
0.8
0.6
0.4
0.2
0.0
Probability
50
100
150
200
250
transmembrane
inside
outside
b
b-i
b-ii
SignalP-5.0 prediction (Eukarya): PbrIDD2
SP(Sec/SPI)
CS
OTHER
0.8
0.4
0.0
Probability
M G E L D N S S S P L P K V S T A S G G E A S L T S S A R E A M T R S E P P Q K K K R N L P G M P D P E A D V I T L S P T T L L A T N R F V
0
20
40
60
Protein sequence
TMHMM posterior probabilities for PbrIDD2
1.2
1.0
0.8
0.6
0.4
0.2
0.0
Probability
50
100
150
200
250
300
350
transmembrane
inside
outside

FIGURE S5

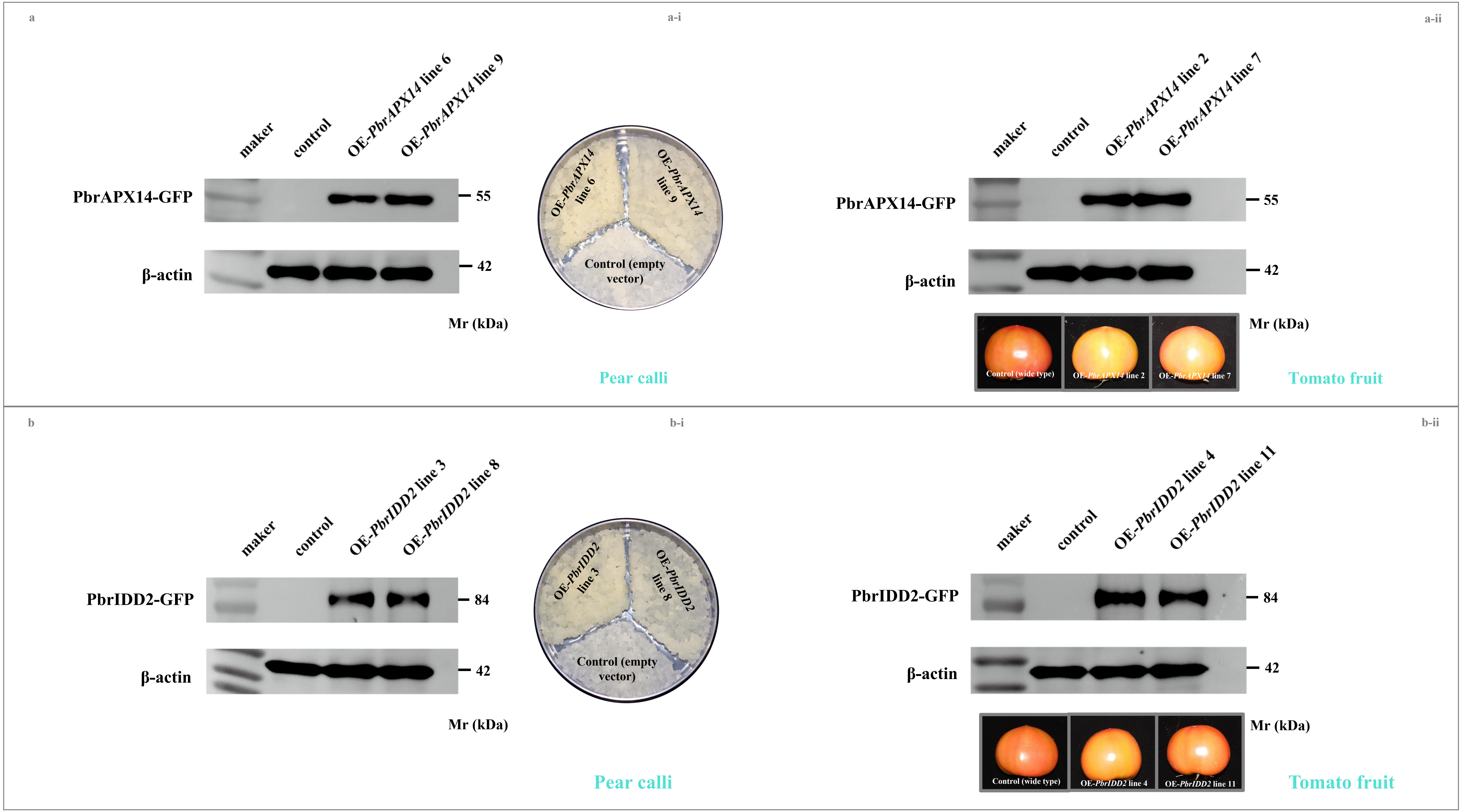
a
a-i
a-ii
maker
control
OE-PbrAPX14 line 6
OE-PbrAPX14 line 9
PbrAPX14-GFP
β-actin
55
42
Mr (kDa)
OE-PbrAPX14 line 6
OE-PbrAPX14 line 9
Control (empty vector)
Pear calli
maker
control
OE-PbrAPX14 line 2
OE-PbrAPX14 line 7
PbrAPX14-GFP
β-actin
55
42
Mr (kDa)
Control (wide type)
OE-PbrAPX14 line 2
OE-PbrAPX14 line 7
Tomato fruit
b
b-i
b-ii
maker
control
OE-PbrIDD2 line 3
OE-PbrIDD2 line 8
PbrIDD2-GFP
β-actin
84
42
Mr (kDa)
OE-PbrIDD2 line 3
OE-PbrIDD2 line 8
Control (empty vector)
Pear calli
maker
control
OE-PbrIDD2 line 4
OE-PbrIDD2 line 11
PbrIDD2-GFP
β-actin
84
42
Mr (kDa)
Control (wide type)
OE-PbrIDD2 line 4
OE-PbrIDD2 line 11
Tomato fruit

FIGURE S6

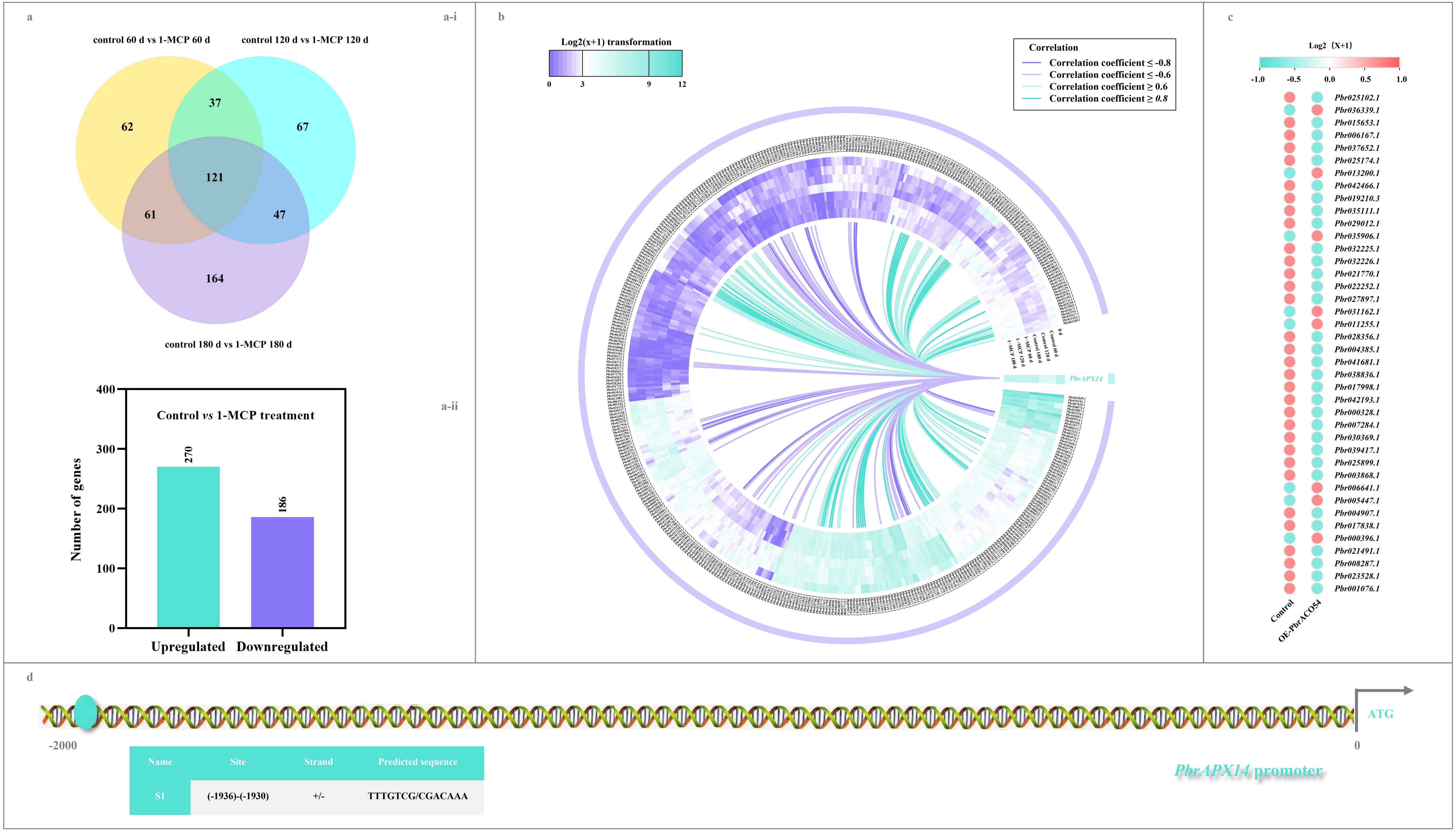
a
a-i
control 60 d vs 1-MCP 60 d
control 120 d vs 1-MCP 120 d
control 180 d vs 1-MCP 180 d
62
37
67
121
61
47
164
a-ii
Control vs 1-MCP treatment
Number of genes
0
100
200
300
400
270
186
Upregulated
Downregulated
b
Log2(x+1) transformation
0
3
9
12
Correlation
Correlation coefficient ≤ -0.8
Correlation coefficient ≤ -0.6
Correlation coefficient ≥ 0.6
Correlation coefficient ≥ 0.8
PbrAPX14
c
Log2（X+1)
-1.0
-0.5
0.0
0.5
1.0
Pbr025102.1
Pbr036339.1
Pbr015653.1
Pbr006167.1
Pbr037652.1
Pbr025174.1
Pbr013200.1
Pbr042466.1
Pbr019210.3
Pbr035111.1
Pbr029012.1
Pbr035906.1
Pbr032225.1
Pbr032226.1
Pbr021770.1
Pbr022252.1
Pbr027897.1
Pbr031162.1
Pbr011255.1
Pbr028356.1
Pbr004385.1
Pbr041681.1
Pbr038836.1
Pbr017998.1
Pbr042193.1
Pbr000328.1
Pbr007284.1
Pbr030369.1
Pbr039417.1
Pbr025899.1
Pbr003868.1
Pbr006641.1
Pbr005447.1
Pbr004907.1
Pbr017838.1
Pbr000396.1
Pbr021491.1
Pbr008287.1
Pbr023528.1
Pbr001076.1
Control
OE-PbrACO54
d
-2000
0
ATG
PbrAPX14 promoter
Name
Site
Strand
Predicted sequence
S1
(-1936)-(-1930)
+/-
TTTGTCG/CGACAAA

FIGURE S7

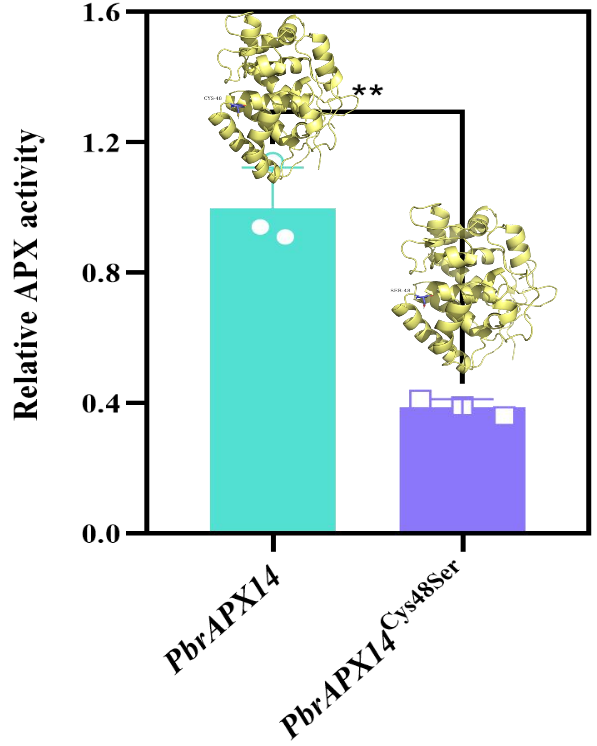

Relative APX activity
1.6
1.2
0.8
0.4
0.0
**
PbrAPX14
PbrAPX14Cys48Ser

FIGURE S8

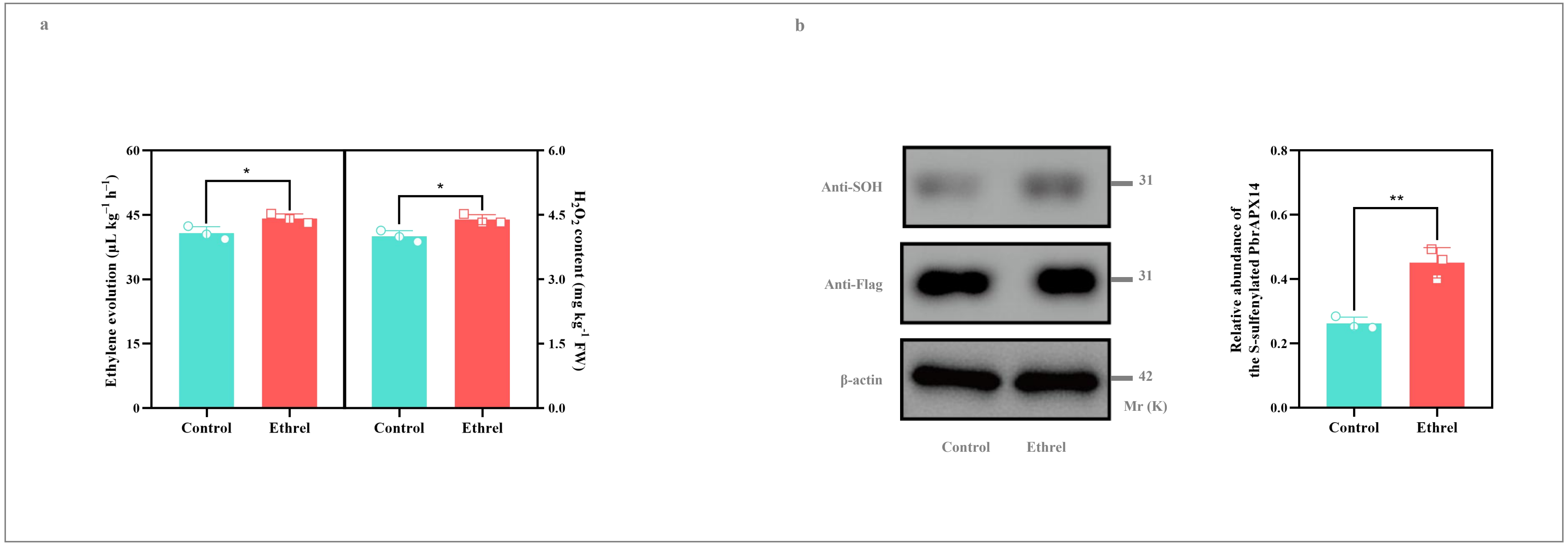

a
b
Ethylene evolution (μL kg⁻¹ h⁻¹)
H₂O₂ content (mg kg⁻¹ FW)
Control
Ethrel
Anti-SOH
Anti-Flag
β-actin
31
31
42
Mr (K)
Relative abundance of the S-sulfenylated PbrAPX14

FIGURE S9

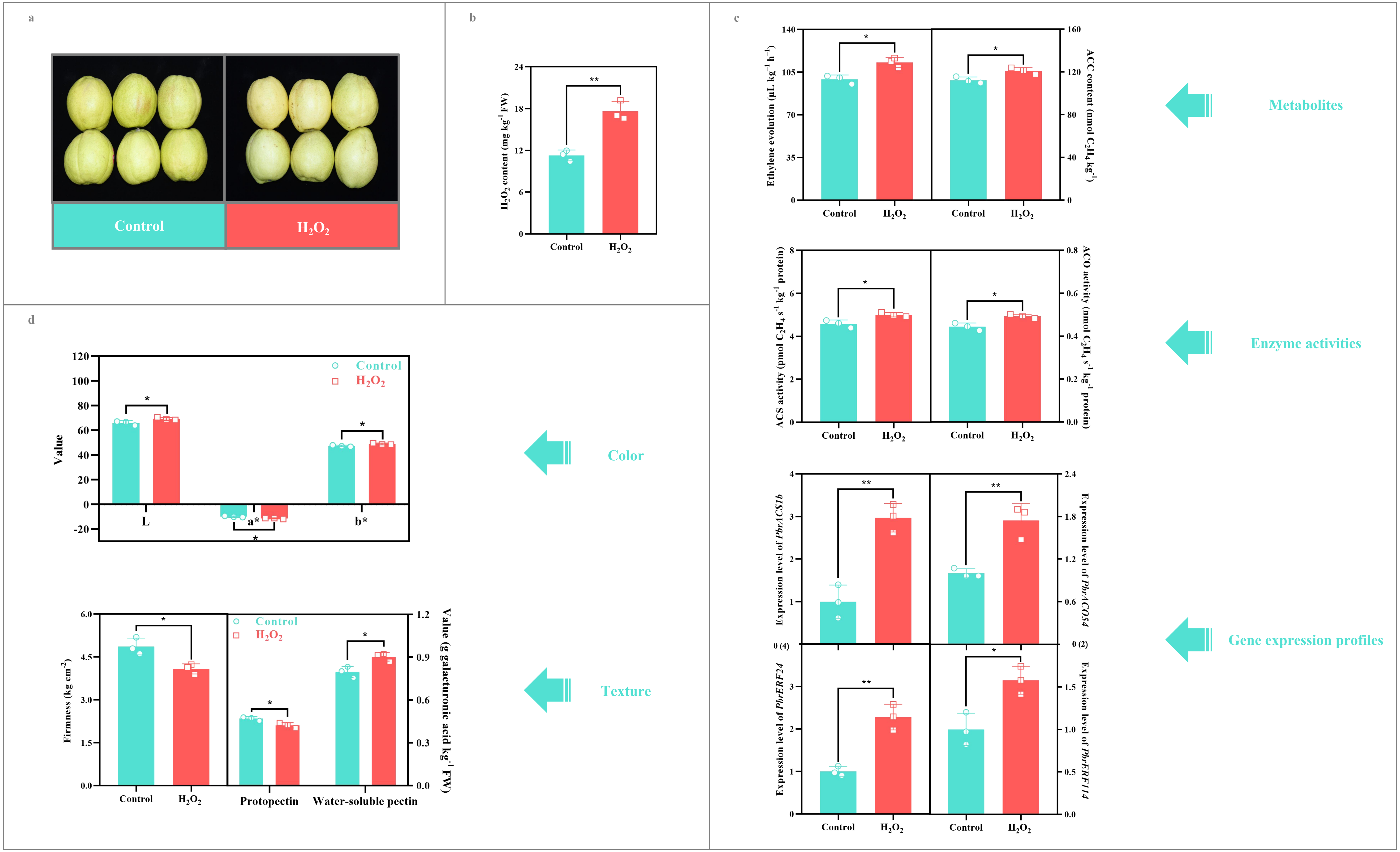

a
Control
H_2O_2
b
H_2O_2 content (mg kg^{-1} FW)
**
Control
H_2O_2
c
Ethylene evolution (μL kg^{-1} h^{-1})
ACC content (nmol C_2H_4 kg^{-1})
Metabolites
ACS activity (pmol C_2H_4 s^{-1} kg^{-1} protein)
ACO activity (nmol C_2H_4 s^{-1} kg^{-1} protein)
Enzyme activities
Expression level of *PbrACS1b*
Expression level of *PbrACO54*
Expression level of *PbrERF24*
Expression level of *PbrERF114*
Gene expression profiles
d
Value
L
a*
b*
Color
Firmness (kg cm^{-2})
Value (g galacturonic acid kg^{-1} FW)
Protopectin
Water-soluble pectin
Texture

FIGURE S10

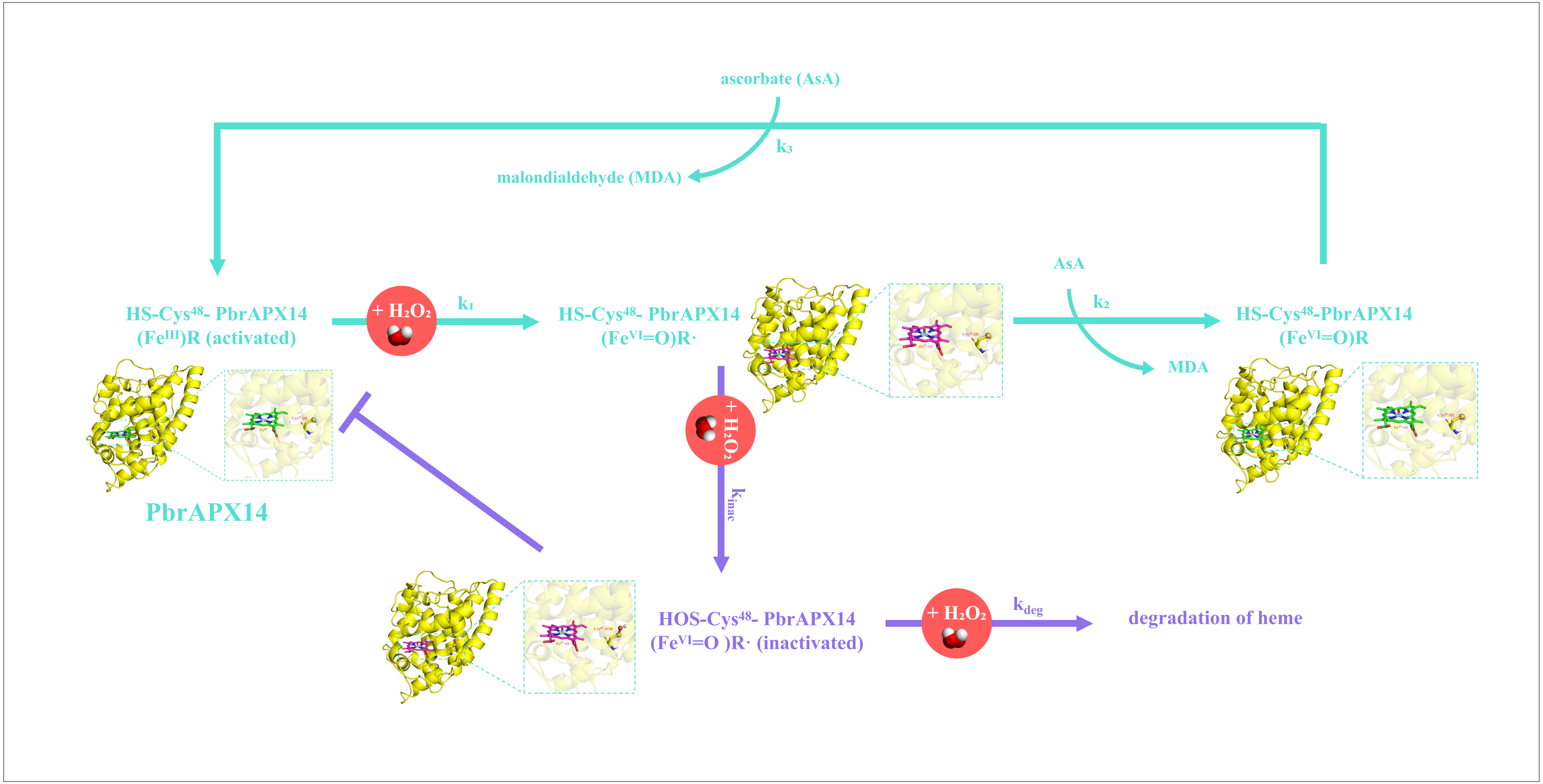
ascorbate (AsA)
k3
malondialdehyde (MDA)
HS-Cys48- PbrAPX14
(FeIII)R (activated)
+ H2O2
k1
HS-Cys48- PbrAPX14
(FeVI=O)R·
AsA
k2
MDA
HS-Cys48-PbrAPX14
(FeVI=O)R
PbrAPX14
+ H2O2
kinac
HOS-Cys48- PbrAPX14
(FeVI=O)R· (inactivated)
+ H2O2
kdeg
degradation of heme

FIGURE S11
